# Obesity Modifies the Proteomic Profile of the Periodontal Ligament

**DOI:** 10.3390/ijms24021003

**Published:** 2023-01-05

**Authors:** Andressa V. B. Nogueira, Maria Eduarda S. Lopes, Camila C. Marcantonio, Cristiane R. Salmon, Luciana S. Mofatto, James Deschner, Francisco H. Nociti-Junior, Joni A. Cirelli

**Affiliations:** 1 Department of Periodontology and Operative Dentistry, University Medical Center of the Johannes Gutenberg University, 55131 Mainz, Germany; 2 Department of Diagnosis and Surgery, School of Dentistry at Araraquara, São Paulo State University—UNESP, Araraquara 14801-903, São Paulo, Brazil; 3 Department of Prosthodontics and Periodontics, Division of Periodontics, Piracicaba Dental School, University of Campinas—UNICAMP, Piracicaba 13414-903, São Paulo, Brazil; 4 Department of Genetics, Evolution, Microbiology, and Immunology, Institute of Biology, University of Campinas—UNICAMP, Campinas 13083-862, São Paulo, Brazil; 5 São Leopoldo Mandic Research Center, Campinas 13045-755, São Paulo, Brazil

**Keywords:** obesity, periodontal ligament, proteomics, prolargin, protein Sec13 homolog, superoxide dismutase

## Abstract

This study aimed to assess the obesity effects on the proteomic profile of the periodontal ligament of rats submitted to obesity induction by a high-fat diet. Eight Holtzman rats were divided into control (n = 3) and obese (n = 5) groups. The maxillae were histologically processed for laser capture microdissection of the periodontal ligament of the first maxillary molars. Peptide mixtures were analyzed by LC-MS/MS. A total of 1379 proteins were identified in all groups. Among them, 335 (24.30%) were exclusively detected in the obese group, while 129 (9.35%) proteins were uniquely found in the control group. Out of the 110 (7.98%) differentially abundant proteins, 10 were more abundant and 100 had decreased abundance in the obese group. A gene ontology analysis showed some proteins related to obesity in the “extracellular exosome” term among differentially identified proteins in the gene ontology cellular component terms Prelp, Sec13, and Sod2. These three proteins were upregulated in the obese group (*p* < 0.05), as shown by proteomic and immunohistochemistry analyses. In summary, our study presents novel evidence that the proteomic profile of the periodontal ligament is altered in experimental obesity induction, providing a list of differentially abundant proteins associated with obesity, which indicates that the periodontal ligament is responsive to obesity.

## 1. Introduction

Obesity is a chronic, complex, and inflammatory disease defined by exaggerated or abnormal fat accumulation that impairs an individual’s health [1,2,3]. In recent years, the prevalence of obesity has increased significantly worldwide. The main cause of obesity is the energy imbalance between calorie intake and expenditure. In addition, other factors contribute to obesity, such as a sedentary lifestyle, genetic factors, hormonal dysfunctions, industrialization, and food production [4]. Body mass index (BMI) has been used to measure obesity and is calculated as the ratio of body weight to height. According to the WHO, a person with a BMI greater than or equal to 30 kg/m^2^ is considered obese. In addition, recognizing the importance of visceral fat as a health risk factor, measurements of waist circumference or waist-to-hip ratio have also been used [5]. These measures are important to evaluate the risk of comorbidities. Thus, increased body weight, as measured by BMI, waist circumference, or waist-to-hip ratio, is a critical risk factor for some non-communicable diseases such as cardiovascular diseases, diabetes, musculoskeletal disorders, and cancer [6,7,8]. Lately, obesity has been considered by the new classification of periodontal diseases and conditions as one of the systemic conditions that negatively influences the periodontal support tissues by influencing the pathogenesis of periodontal diseases [9]. The pathomechanistic link between obesity and other chronic diseases may be due to the low-grade systemic inflammation in obesity. Thus, many adipokines are produced not only in the adipose tissue by adipocytes but also by other cells and tissues during obesity. Interestingly, adipokines have also been found in periodontal ligament (PDL) cells and tissues [10,11,12,13].

The periodontium is the tooth-supporting tissue comprised of the gingiva, PDL, root cementum, and alveolar bone. The PDL is a specialized connective tissue that attaches the root cementum to the alveolar bone [14]. It is essential in attenuating the occlusal stresses through the transmission and absorption of mechanical stresses. Interestingly, the PDL has a strong and flexible three-dimensional structure capable of tolerating multiaxial loading and functioning properly under different forces such as tension, compression, shear, and torsion [15]. Furthermore, PDL is a highly vascularized and cellularized tissue, containing many fibroblasts, a high metabolic rate, and cellular turnover [14]. Its capacity for regeneration has been investigated, as it contains undifferentiated mesenchymal stem cells [16].

Proteins are produced under physiological and pathological conditions through the metabolism of cells and tissues in the human body. In the last three decades, proteomic analysis has been used extensively. It involves a large-scale study of proteins based on the systematic identification and quantification of the complete proteome of a biological system such as cells, tissues, and organs. The protein abundance through proteomics enables the identification of the main proteins present in a sample and also differentially abundant proteins in different samples. Lately, proteomic analysis has been used widely in different challenged oral cells, tissues, and fluids, such as enamel [17], pulp [18,19], dental cementum [20,21,22], PDL [23,24], alveolar bone [22], periodontal ligament cells [25,26], gingival fibroblasts [27], gingival tissue [28,29], saliva [30], and gingival crevicular fluid [31], enabling researchers to better understand biological processes. Therefore, characterizing the PDL protein profile in obesity conditions becomes important in identifying possible biological markers different from those in health conditions. In this context, the present study was designed to assess the effect of obesity on the proteomic profile of the PDL in rats for the first time.

## 2. Results

### 2.1. General Proteomic Profile of PDL in the Control and Obese Groups

**Figure 1:**
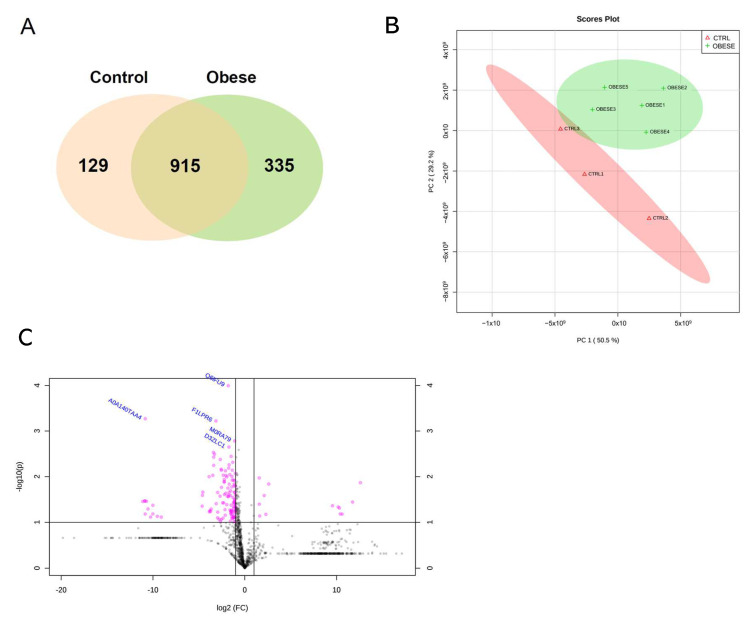
Summary of the proteomic profile of PDL in the control and obese groups. (**A**) Distribution of the total 1379 proteins identified in both groups (915) and exclusively in the control (129) and obese (335) groups, as shown by Venn diagram. (**B**) Difference between the samples of the control (CTRL) and obese groups and the similarity between the samples within each group, as exhibited by PCA. (**C**) Distribution of significantly differentially regulated proteins (*p* < 0.05) in the PDL of the control (left side) and obese (right side) groups determined by the beta-binomial statistical test (*p* < 0.05), as illustrated by a volcano plot.

A total of 1379 proteins were identified in all groups and their distribution. Among the identified proteins, 335 (24.30%) were exclusively detected in the obese group, while 129 (9.35%) proteins were uniquely found in the control group. In addition, 915 (66.35%) proteins were commonly identified in both the control and obese groups (Figure 1A). A principal component analysis (PCA) was performed to transform and group the datasets of the control and obese groups in an unsupervised method (Figure 1B). The analysis revealed that the first principal component (PC1) presented 50.50% of the variance within the dataset, whereas the second principal component (PC2) showed 29.20%. In other words, the rat samples with similar protein profiles were clustered together, leading to two different clusters of the two experimental groups, showing a distinction between the proteomes of the control and obese groups. The volcano plot shows the differential abundance of proteins between the control and obese groups (Figure 1C). Proteins with a 1.5-fold difference and statistical significance (*p* < 0.05) were plotted. The control group presented a higher amount of significantly abundant proteins compared to the obese group. Of the total proteins identified, the abundance of 110 (7.98 %) proteins was significantly (*p* < 0.05) altered, 10 were more abundant, and 100 had a decreased abundance in the obese group (Table S1). Among those ten proteins more abundant in the obese group, five were exclusive: Prelp, Arpc5, I6l9g6, Prof2, and A3fm27 (*p* < 0.05). From the 100 proteins more abundant in the control group, six were exclusive: Gmppa, B2rza9, A0a140taa4, M0rd20, Q63002, and Cpsf5 (*p* < 0.05). 

### 2.2. Differentially Identified Proteins in PDL

**Figure 2:**
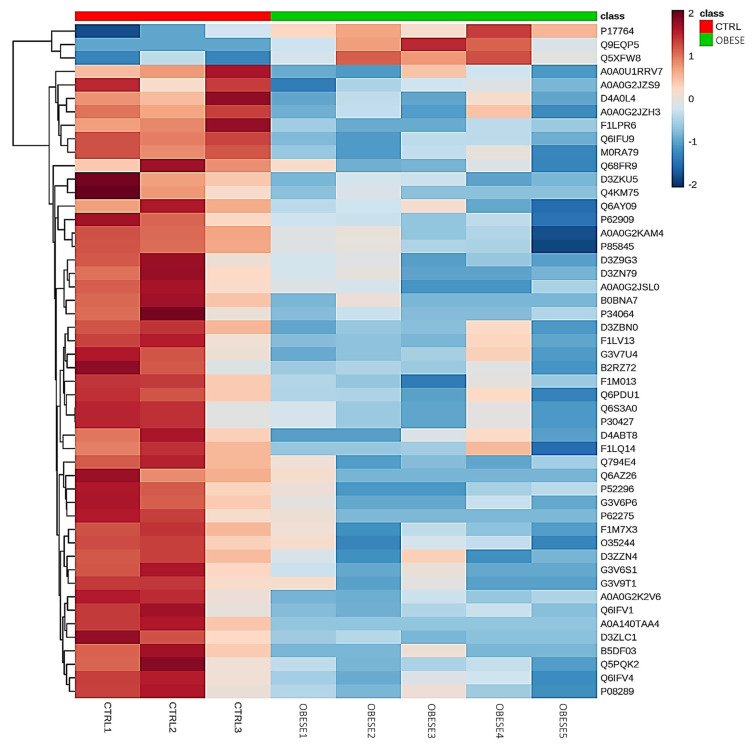
Hierarchical clustering of the top 50 proteins of increased and reduced abundance in the control (CTRL) and obese groups, as shown by the heat map. Data were classified by *p*-value (*p* < 0.05) and estimated using the Z-score calculation on Log2 spectral count values, applying the Euclidean distance and average linkage method.

Hierarchical clustering of the top 50 differentially abundant proteins showed a distinction between the control and obese groups in a heat map analysis (Figure 2). 

**Figure 3:**
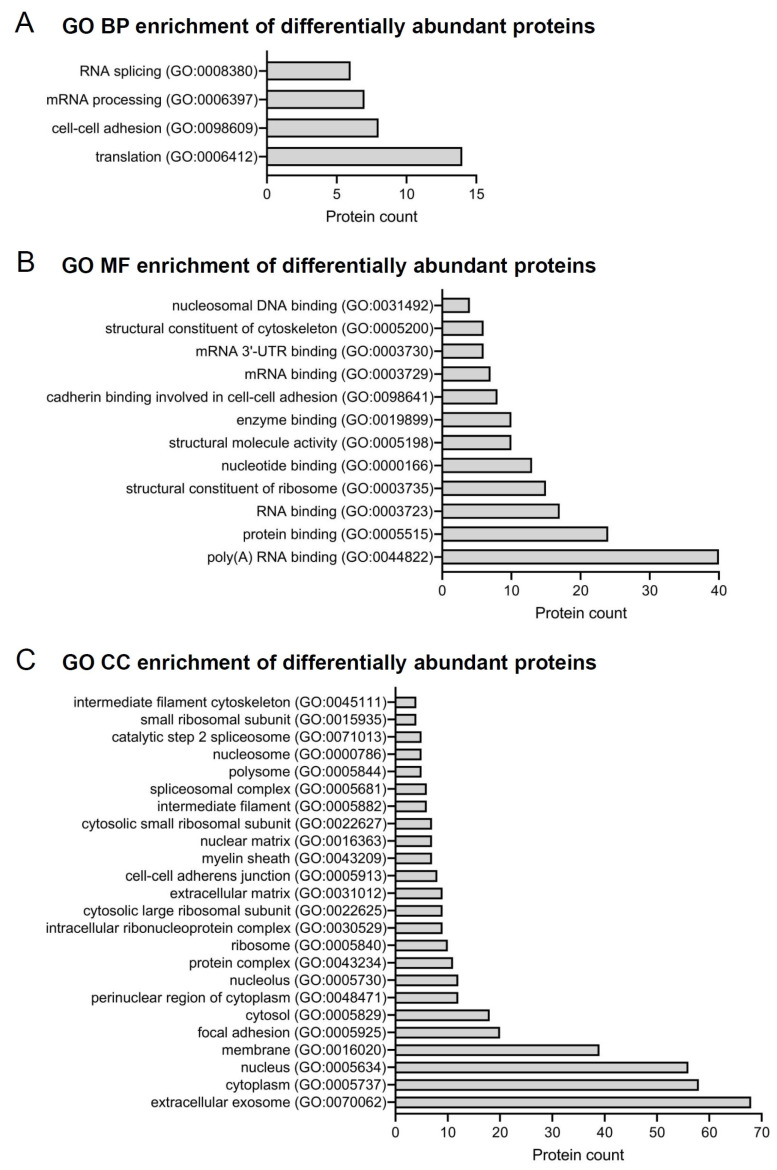
GO analysis of all differentially abundant proteins identified in the PDL of the control and obese groups. (**A**) Graph representing the differentially detected proteins in the GO BP terms related to “translation” (GO:0006412), “cell-cell adhesion” (GO:0098609), “mRNA processing” (GO:0006397), and “RNA splicing” (GO:0008380). (**B**) Graph demonstrating the differentially abundant proteins in the GO MF terms especially related to “poly(A) RNA binding” (GO:0044822), “protein binding” (GO:0005515), “RNA binding” (GO:0003723), and “structural constituent of ribosome” (GO:0003735). (**C**) Differentially, proteins were detected in the GO CC terms, which were the largest category of differentially identified proteins, as they were related to 24 enriched terms, such as “extracellular exosome” (GO:0070062), “cytoplasm” (GO:0005737), “nucleus” (GO:0005634), and “membrane” (GO:0016020). The number of proteins associated with each GO enrichment term is shown.

A Gene Ontology (GO) enrichment analysis using the Database for Annotation, Visualization, and Integrated Discovery platform (DAVID v 6.8, accessed on 15 May 2019 https://david.ncifcrf.gov) was performed on the proteins differentially identified in the control and obese groups to evaluate the functional meaning of the results. The proteins were analyzed according to the GO terms (level 2): cellular component (CC), molecular function (MF), and biological process (BP). As shown in Figure 3A, the differentially detected proteins in the GO BP terms were related to “translation” (GO:0006412), “cell-cell adhesion” (GO:0098609), “mRNA processing” (GO:0006397), and “RNA splicing” (GO:0008380). In addition, in the GO MF terms, there were 12 significant terms and the majority of differentially identified proteins were involved with “poly (A) RNA binding” (GO:0044822), “protein binding” (GO:0005515), “RNA binding” (GO:0003723), and “structural constituent of ribosome” (GO:0003735) (Figure 3B). The GO CC terms were the largest category of differentially identified proteins, which were related to 24 enriched terms, such as “extracellular exosome” (GO:0070062), “cytoplasm” (GO:0005737), “nucleus” (GO:0005634), and “membrane” (GO:0016020) (Figure 3C).

Among the differentially identified proteins in the GO CC terms, there are some proteins related to obesity in the GO term “extracellular exosome”, as shown in Table 1, including prolargin (Prelp), protein Sec13 homolog (Sec13), and superoxide dismutase (Sod2). Prelp, Sec13, and Sod2 were significantly more abundant in the obese group (*p* < 0.05).

**Figure 4:**
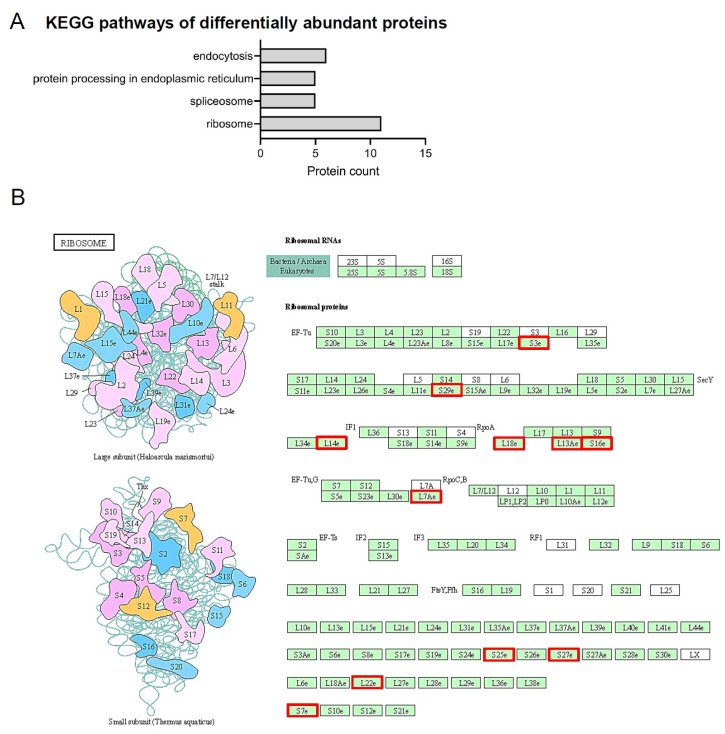
Analysis of all differentially abundant proteins in the PDL of the control and obese groups. (**A**) KEGG pathways of differentially abundant proteins and the number of proteins of each KEGG pathway term. (**B**) Ribosomal pathway information generated by DAVID software. Red squares represent the eleven ribosomal proteins that were more abundant in the control group compared to the obesity group.

**Figure 5:**
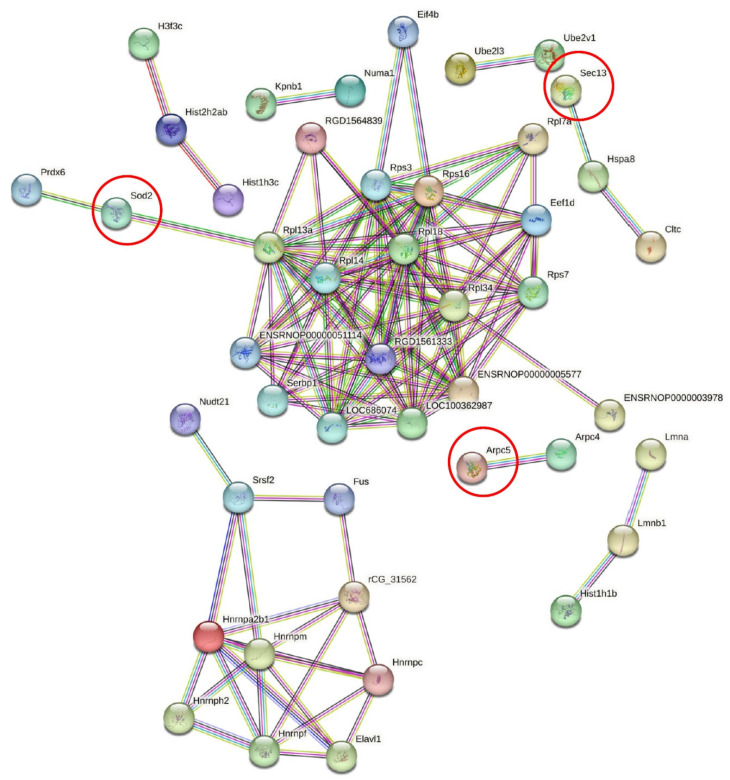
Protein-protein interaction network analysis (high-confidence of 0.700 with hidden disconnected nodes in the network) of the differentially abundant proteins in the PDL of the control and obese groups. Red circles represent three proteins that were more abundant in the obesity group compared to the control group: Sod2, Sec13, and Arpc5.

The KEGG pathways of differentially abundant proteins were analyzed by DAVID software and the following pathways were identified: “ribosome”, “spliceosome”, “protein processing in the endoplasmic reticulum”, and “endocytosis” (Figure 4A). Only the “ribosome” pathway was statistically (*p* < 0.05) significant, and a detailed expression of the 11 proteins of this pathway is shown in Table S1. In addition, the complete ribosome pathway and a gene report with these 11 proteins are shown in Figure 4B and Figure S1. The ribosomal proteins of the ribosome pathway were all more abundant in the control group compared to the obesity group. Protein-protein interactions of differentially abundant proteins in the PDL of the control and obese groups were assessed using the Protein-Protein Interaction Networks Functional Enrichment Analysis platform (STRING v 11.5, released 12 August 2021, accessed on 6 December 2022: https://string-db.org), as illustrated in Figure 5. A high confidence level of 0.700 was used and a strong protein-protein interaction could be observed. Sod2, Sec13, and Actin-related protein 2/3 complex subunit 5 (Arpc5) were the most abundant proteins in the obese group compared to the control group, and had a strong interaction with some proteins. Sod2, for example, showed a strong interaction with Peroxiredoxin 6 (Prdx6), and both are antioxidant enzymes. Furthermore, Sod2 strongly interacted with several ribosomal proteins (Rpl). In addition, Sec13 showed strong interactions with Ubiquitin-conjugating enzyme E2 L3 (Ube2l3), Ubiquitin-conjugating enzyme E2 variant 1 (Ube2v1), 60S ribosomal protein L7-A (Rpl7a), Heat shock cognate 71 kDa protein (Hspa8), and Clathrin heavy chain 1 (Cltc). Proteins lacking interactions did not appear in the network.

### 2.3. Immunolocalization of the Selected Proteins Prelp, Sec13, and Sod2 in PDL

**Figure 6:**
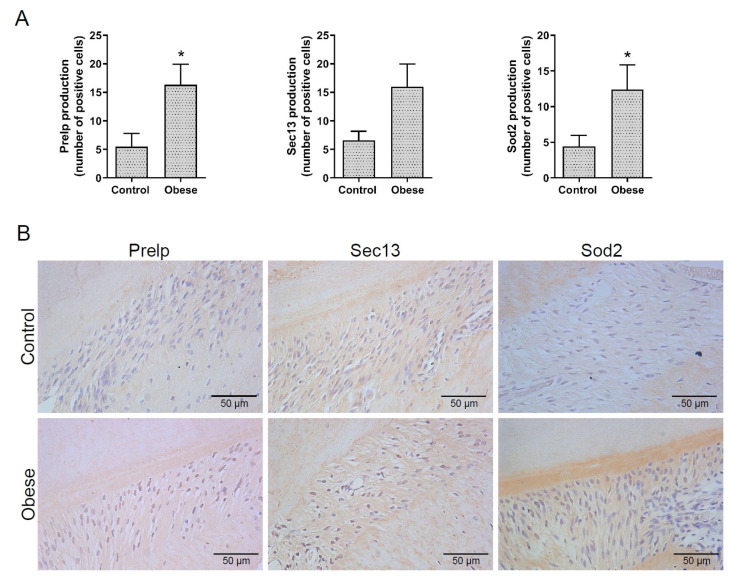
(**A**) Graphs with mean and standard deviation showing the number of cells positive to Prelp, Sec13, and Sod2, as analyzed by IHC staining (*p* < 0.05). * Significant (*p* < 0.05) difference. (**B**) Representative images (400× magnification) of IHC staining for Prelp, Sec13, and Sod2 proteins, respectively, in tissue sections from the control and obese groups.

The selected proteins Prelp, Sec13, and Sod2 identified by the proteomic analysis were chosen for their relevant role in obesity and different abundance between groups. Their presence was confirmed in periodontal tissues by immunohistochemistry (IHC) staining. An increased number of positive cells to Prelp, Sec13, and Sod2 proteins was observed in the obese group compared to the control group, but only the expression of Prelp and Sod2 was statistically significant (*p* < 0.05). The IHC analysis confirms the proteomic analysis (Figure 6A,B).

### 2.4. Exclusively Identified Proteins in PDL 

**Figure 7:**
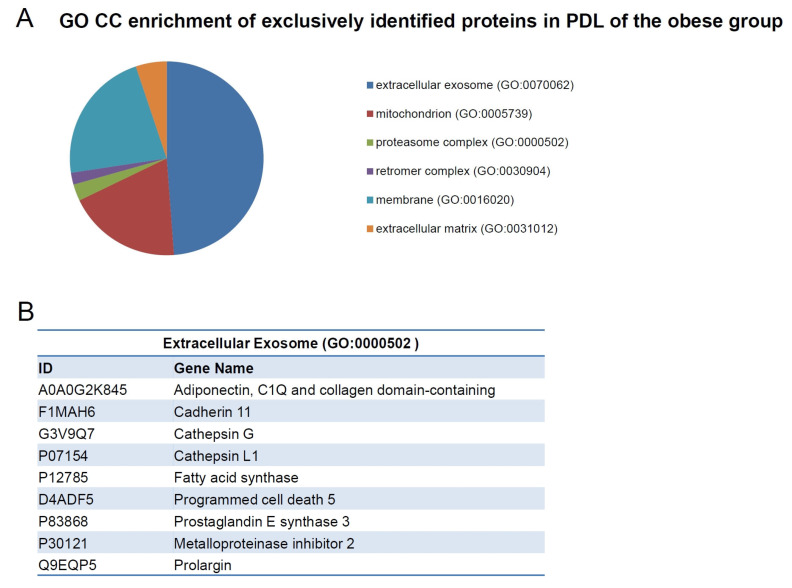
(**A**) Graph containing the GO enrichment analysis of exclusively identified proteins in the PDL of obese animals using DAVID software, which revealed that the proteins were related to the term “cellular component” (CC). (**B**) List of some proteins detected in the GO CC term “extracellular exosome” are highlighted: adiponectin (A0A0G2K845), cadherin 11 (F1MAH6), cathepsin G (G3V9Q7), cathepsin L1 (P07154), fatty acid synthase (P12785), programmed cell death 5 (D4ADF5), prostaglandin E synthase 3 (P83868), metalloproteinase inhibitor 2 (P30121), and prolargin (Q9EQP5).

**Figure 8:**
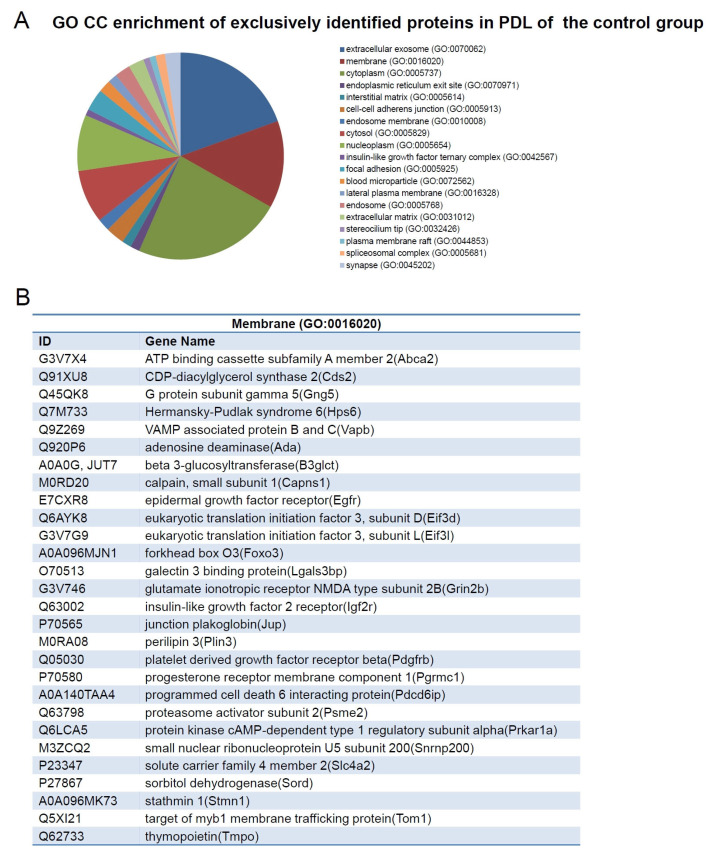
(**A**) Graph containing the GO enrichment analysis of exclusively identified proteins in PDL of control animals using DAVID software, which revealed that the proteins were related to the term “cellular component” (CC). (**B**) List of the proteins of the GO CC term “membrane”.

GO enrichment analysis in exclusively identified proteins in the PDL of obese and control rats revealed that proteins were related to GO CC terms (Figure 7A and Figure 8A). The most enriched GO CC terms were determined by a *t*-test followed by the Benjamini-Hochberg test with *p* < 0.05. There were six terms; among them, five terms were significant (*p* < 0.05) in the GO CC terms of the obese group, which were: “extracellular exosome” (GO:0070062) with 123 proteins, “mitochondrion” (GO:0005739) with 48 proteins, “proteasome complex” (GO:0000502) with seven proteins, “retromer complex” (GO:0030904) with five proteins, and “membrane” (GO:0016020) with 56 proteins (Figure 7A). Among the proteins detected in the GO term “extracellular exosome”, we can highlight adiponectin (A0a0g2k845), cadherin 11 (F1mah6), cathepsin G (G3v9q7), cathepsin L1 (P07154), fatty acid synthase (P12785), programmed cell death 5 (D4adf5), prostaglandin E synthase 3 (P83868), metalloproteinase inhibitor 2 (P30121), and prolargin (Prelp) (Figure 7B). In the control group, there were 19 terms, but only 3 significant (*p* < 0.05) enriched GO CC terms, which were: “extracellular exosome” (GO:0070062) with 40 proteins; “membrane” (GO:0016020) with 28 proteins; and “cytoplasm” (GO:0005737) with 48 proteins (Figure 8A).

## 3. Discussion

The present study provides novel evidence that obesity affects the PDL at a molecular level. Our results reveal a critical difference in the proteomic profile between the PDL of the obese and control groups. Moreover, among the differentially abundant proteins, most of the proteins that were more abundant in the control group had decreased abundance in the obese group, suggesting that important molecules could mediate the detrimental effects of obesity on periodontal tissues, such as PDL. 

Obesity is defined as abnormal or excessive fat accumulation, and has become an increasing public health problem worldwide [1,3]. As a consequence of obesity, systemic inflammation occurs [32]. Thus, not only adipose cells but also other cells and tissues in the body are responsible for regulating inflammation and immunity associated with the dysregulated release of a variety of pro-inflammatory and anti-inflammatory molecules, such as adipokines, cytokines, and chemokines [33]. This inflammatory state present in obesity may act as a pathomechanistic link between obesity and other non-communicable diseases such as cardiovascular diseases, diabetes, musculoskeletal disorders, cancer, and periodontitis [6,7,8,34]. Interestingly, our group has recently demonstrated that obesity modifies the proteomic profile of PDL of rats subjected to experimental periodontitis, suggesting a critical effect of obesity on the PDL response to inflammation and tissue remodeling. Our study showed that obesity modulated the abundance of proteins related to periodontitis, such as spondin1, tartrate-resistant acid phosphatase, and vinculin [23]. Moreover, in another study, we observed the detrimental effects of obesity on the protein abundance of the PDL of rats exposed to orthodontic movement. It suggested that the chronic inflammation in obesity might interfere with the aseptic inflammation induced by the mechanical loading by the increase of vinculin, osteopontin, and cathepsin D [24]. Therefore, it is exciting to see how proteomic analysis enables us to observe that a systemic condition or disease can interfere with a microenvironment, such as PDL.

The GO term mostly found by the GO enrichment analysis and, consequently, the one that obtained the highest number of proteins was the CC term “extracellular exosome” (GO:0070062). Also known as exosomes, extracellular exosomes are extracellular vesicles produced and released by most cells. They have different functions, and are involved in intracellular regulatory processes such as cellular homeostasis and autophagy, for example Furthermore, they act as mediators in intercellular communication, immune responses, and infection. Interestingly, exosomes are considered as potential biomarkers for the diagnosis and treatment of various diseases [35]. Exosomes in obesity become altered with different miRNAs and enriched with molecules involved in inflammation, immune competence, and cellular activation. Furthermore, they are released by adipocytes, which can induce a pro-inflammatory state in adipose tissue and promote insulin resistance [36].

A KEGG pathway analysis of the differentially abundant proteins revealed an enrichment of the ribosome pathway. The eleven proteins found in this pathway significantly decreased in their abundance in the obese group compared to the control group, showing that obesity affects the function of ribosomes. Ribosomal proteins are responsible for the translational process of cells and act in ribosomal biogenesis. They are key players during normal cell physiology, such as cell growth, proliferation, and development. They play an important role in cellular responses to internal and external stimuli and the pathogenesis of diseases. Ribosomal proteins have been demonstrated to regulate gene expression, the translation of proteins, and the cell cycle. Any dysregulation of the ribosome biogenesis can result in atypical cell proliferation and clinical manifestations of diseases and conditions such as cancer and metabolic disorders. In obesity, cellular functions are required due to the hyperplasia and hypertrophy of adipocytes, and are dependent on increased ribosomal proteins [37]. Furthermore, there is an increased metabolic demand in obesity, which could enhance ribosomal expression due to the increased protein synthesis in blood cells [38]. This evidence shows that the ribosomal pathway is upregulated in obese subjects, as reported in the whole-genome expression of whole blood [37,38]. However, corroborating our result, we found some studies [39,40,41] that observed a negative correlation between the ribosome pathway and obesity. Remarkably, a previous proteomic analysis of human skeletal muscle demonstrated a reduced abundance of proteins related to ribosome activity in obesity. Surprisingly, bariatric surgery could reverse the negative effects of obesity by increasing the expression of ribosome proteins [39]. Van der Kolk et al. [41] observed the downregulation of the ribosome pathway in adipose tissue and in the skeletal muscle of heavier co-twins. Unfortunately, these studies do not comment on the role of the ribosome pathway in obesity. The 11 ribosomal proteins of the ribosome pathway found in the present study are localized in the cell cytoplasm and could perform different extraribosomal function(s). More studies are necessary to better comprehend the role of ribosomal proteins in obesity.

Proline/arginine-rich end leucine-rich repeat protein (Prelp) is a protein present in the extracellular matrices of collagen-rich tissues. [42] It has been demonstrated to be expressed in the ligaments, cartilage, bone matrices, tendon, skin, sclera, lungs, and heart [43,44,45]. Prelp is responsible for anchoring basement membranes to the underlying connective tissues [43]. It has been reported that Prelp reconstructs ruptured ligament tissue through the differentiation of stem cells into ligament tissue [46]. Furthermore, Prelp is involved in bone metabolism, as the N-terminal peptide of Prelp has been shown to inhibit osteoclast differentiation and activity in addition to bone loss [47]. In our study, as assessed by proteomic analysis and IHC, the production of Prelp was significantly increased in the PDL of high-fat diet-induced obesity rats compared to the control group. A study recently reported that Prelp expression is increased in adipocytes of obese mice. Interestingly, in this same study, they developed adipocyte-specific Prelp transgenic mice and could observe that the overexpression of Prelp resulted in considerable adipose tissue fibrosis and insulin resistance. Their results suggested that this protein may play an important role in obesity concerning adipose tissue remodeling [48]. Furthermore, Prelp belongs to the small leucine-rich repeat proteoglycans (SLRPs). Evidence has shown that SLRPs are involved in insulin resistance related to visceral secretome. In addition, they positively correlate with BMI and central obesity and mediate metabolic inflammation, suggesting that extracellular matrix components are critical to the pathogenesis of obesity [49].

Superoxide dismutases (Sods) are a family of enzymatic antioxidants that neutralize superoxide anions. The isozyme Sod2 is mainly found in mitochondria but may also be located in the extracellular compartment [50]. Sod2 is involved in the response of periodontal cells and tissues exposed to bacterial and mechanical signals [51,52,53,54]. Furthermore, evidence shows an association between obesity, increased reactive oxygen species (ROS), and intracellular oxidative stress. Therefore, the antioxidant system is necessary to eliminate ROS or minimize their harmful effects. Our results show that the Sod2 protein was significantly more abundant in the obese group compared to the control group, probably due to the increase in the number of superoxide anions, suggesting a preventive role of Sod2 in inflammation progression that occurred as a result of obesity. A previous study that focused on the presence of Sod2 in saliva showed a greater total amount of Sod2 in subjects with obesity than in healthy controls [55]. Regarding the evaluation of Sod2 expression in the adipose tissue of obese children, Sod2 was significantly increased, whereas its activity was reduced [56]. This finding suggests that obesity impairs the function of PDL in relation to protein synthesis/secretion.

The protein Sec13 is encountered in the nuclear pore complex (NPC) inside the nucleus, and is a structural component of the coat protein II (COPII) coated vesicles in the cytoplasm [57]. It is involved in the transport of protein from the endoplasmic reticulum to the Golgi apparatus [58]. In inflammation, Sec13 modulates the expression of key immune factors [57]. Food intake in high-fat mice has been shown to upregulate the expression of Sec13 in the hypothalamus [59]. This observation corroborates our results that higher levels of Sec13 were observed in the PDL of the obese group as compared to the healthy control group.

Evaluating protein-protein interactions using STRING analysis, we could observe that Sod2 has a strong interaction with Prdx6. This protein belongs to the peroxiredoxin family, which are antioxidant enzymes capable of catalyzing the reduction of hydrogen peroxide (H_2_O_2_). In addition to its peroxidase activity, Prdx6 has phospholipase A_2_ activity and can hydrolyze phospholipids. The absence of Prdx6 was shown to be related to a higher level of pro-inflammatory markers in the liver, skeletal muscle, and white adipose tissue. Furthermore, in this study, the authors observed that mice lacking Prdx6 were more likely to develop severe liver dysfunction [60]. Corroborating the link between Prdx6 and inflammation, pancreatic beta cells exposed to TNF-alpha and/or IFN-gamma decreased the level of Prdx6 protein [61]. In the present study, Prdx6 was significantly decreased in the obese group compared to the control group, showing that this antioxidant enzyme has an important role in the harmful effects of obesity in some tissues, such as PDL. Future studies must further explore the protein-protein interaction network and its impact on clinical conditions.

In this context, this study contributed by highlighting PDL proteins affected by obesity, suggesting that future clinical studies should investigate these proteins in human oral fluids and in vitro or pre-clinical studies to unravel the mechanisms of their involvement in periodontal health and disease conditions. 

A limitation of the present study is that not all significantly abundant proteins found by proteomic analysis could be validated by IHC analysis. Future studies should be performed to validate these proteins and to better explore their role in obesity.

Taken together, our results clearly show the effects of obesity on relevant GOs, such as the GO CC term “extracellular exosome”. Furthermore, the present study exhibits novel evidence that the PDL is affected by obesity at a molecular level. Moreover, the proteomic profile of PDL is altered in experimental obesity induction, providing a list of differentially abundant proteins associated with obesity, which indicates that PDL is responsive to obesity and that critical molecules might mediate the harmful effects of obesity on periodontal tissues.

## 4. Materials and Methods

### 4.1. Experimental Protocol

The Ethical Committee on Animal Experimentation of the Sao Paulo State University-UNESP, School of Dentistry at Araraquara, Brazil (protocol number 16/2015) approved the experimental protocol. A total of eight male adult Holtzman rats with an average weight of 300 g were kept in the animal facility of the university in plastic cages, under controlled temperature (22–25 °C), and exposed to a 12-h light/dark cycle. The animals received a standard laboratory diet (Labina/Purina, Ribeirão Preto, Brazil) or a high-fat diet in addition to water ad libitum. They were randomly divided into two experimental groups: control animals (n = 3) and obese animals (n = 5), which comprised animals subjected to obesity induction by a high-fat diet, as previously described [23,24]. This high-fat diet had about 3.82 kcal/g and consisted of a mixture of standard rat chow and milk chocolate, peanuts, and sweet biscuits in a 3:2:2:1 ratio and contained 20 g of protein, 20 g of total fat, 48 g of carbohydrates, and 4 g of fiber per 100 g of diet. In contrast, the standard diet contained approximately 2.25 kcal/g and 22 g of protein, 48 g of carbohydrate, 4 g of total fat, 8 g of fiber, and 200 mg of sodium per 100 g of diet. The high-fat diet was replaced daily. After 90 days, all animals were euthanized by anesthetic overdose and the maxillae were collected.

### 4.2. Laser Capture Microdissection (LCM) and Protein Extraction

As previously described [21], the maxillae were histologically processed to obtain serial sections in a buccolingual direction. They were fixed in 10% buffered formalin (Fisher Diagnostics, Middletown, VA, USA) at 4 °C for 24 h, rinsed three times in phosphate-buffered saline (PBS, pH 7.4, Applied Biosystems, Foster City, CA, USA) for 30 min at 4 °C, and decalcified in 20% ethylenediamine tetraacetic acid (EDTA, Merck, Millipore) for 30 days at 4 °C under agitation. The decalcified maxillae were washed three times in PBS for 30 min at 4 °C, dehydrated in three changes of 100% ethanol for 30 min at 4 °C, diaphanized in two changes of xylene for 20 min at room temperature (RT), and, finally, embedded in paraffin and stored at −20 °C. Serial 5 μm thick buccolingual sections of the first molar were obtained and mounted on polyethylene naphthalate membrane glass slides (PEN, Applied Biosystems). Sections were deparaffinized in two changes of xylene for 2 min and one change for 5 min, and then air dried for 5 min. Unstained sections allowed the visualization of the periodontal ligament architecture and were immediately used for microdissection. PDL was microdissected, as previously described [62]. Eight to ten histological sections per sample were microdissected, and the total captured area was calculated and used to normalize the amount of tissue/protein for LC-MS/MS analysis. Caps containing the captured tissues were incubated with 30 μL of 8 M urea for 30 min at RT for protein extraction. Samples were sonicated and centrifuged briefly. Whole protein extracts were reduced by incubation in 5 mM dithiothreitol, alkylated with 14 mM iodoacetamide, and trypsin digested (2 μg) as described previously [21]. The resulting tryptic peptide samples were equilibrated to pH 2.0 with formic acid. Peptide mixtures were desalted and purified using microcolumns ZipTip^®^ C18, P10 Pipette Tips (Merck Millipore, Billerica, MA, USA), dried in a vacuum concentrator, and reconstituted in 0.1% formic acid for analysis.

### 4.3. LC-MS/MS and Bioinformatics Analysis

A mass spectrometry analysis was performed in the BIOMASS Core Facility for Scientific Research of the University of São Paulo, Brazil. Peptide mixtures were analyzed on an LTQ-Orbitrap Velos ETD (Thermo Scientific, Waltham, MA, USA) mass spectrometer coupled with an Easy NanoLC II (Thermo Scientific) ion source. The peptides were separated by a 2–95% acetonitrile gradient in 0.1% formic acid using a C18 PicoFrit analytical column (C18 PepMap, 75 µm id × 10 cm, 3.5 µm particle size, 100 Å pore size; New Objective, Ringoes, NJ, USA) at a flow rate of 300 nL/min for 105 min. The LTQ-Orbitrap Velos was operated in positive ion mode with a data-dependent acquisition. The full scan was obtained in the Orbitrap, with an automatic gain control (AGC) target value of 10^6^ ions and a maximum fill time of 500 ms. Each precursor ion scan was acquired at a resolution of 60,000 FWHM in the 400–1500 *m*/*z* mass range. Peptide ions were fragmented by CID MS/MS using a normalized collision energy of 35. The 20 most abundant peptides were selected for MS/MS and dynamically excluded for 30 s. The samples were analyzed in two technical replicates combined to perform the database search. All raw data were accessed in Xcalibur software (Thermo Fisher Scientific) and processed using Proteome Discoverer (version 1.4.0.288, Thermo Finnigan, San Jose, CA, USA). The MS/MS spectra (msf) generated from raw files were searched against the UniProtKBSwiss-Prot Protein Database (released 2017_01 of 18 January 2017; 553,474 entries), assuming trypsin digestion with parameters set to a maximum of two missing cleavages, with a parent ion tolerance of 20.0 ppm for the MS search and a fragment ion mass tolerance of 0.6 Da. Oxidation of methionine (+16 Da) was set as a dynamic modification, and carbamidomethylation in cysteine residues (+57 Da) was set as a static modification. For label-free protein quantification, the data files were analyzed in MaxQuant software. The quantitative value (normalized spectral counts) was obtained with the protein thresholds set at a minimum 99% probability to achieve a false discovery rate (FDR) < 1% and containing at least one peptide with thresholds established at a minimum 60% probability, XCorr cutoffs +1 > 1.8, +2 > 2.2, +3 > 2.5, and +4 > 3.5. The resulting peak area values were used to analyze the distribution of identified proteins throughout the samples. An independent samples t-test was applied to test for differences in protein intensities between the control and obese sample groups. Protein ratios were calculated from the average peak area values of the control and obese groups. A PCA was performed to determine sample variability between the proteome profiles of control and obese PDLs. A PCA analysis, volcano plot and a heat map graphic organization were performed using MetaboAnalist software (https://www.metaboanalyst.ca/home.xhtml, accessed on 7 June 2018). A GO enrichment analysis and KEGG pathways assignment were performed using DAVID software. The differential abundance of proteins was determined by a *t*-test followed by the Benjamini-Hochberg multiple test correction. The fold-change between differentially regulated proteins was also determined. All statistical tests were calculated using a *p* < 0.05, and only statistically significantly enriched GO terms were reported. Furthermore, the protein-protein interactions of differentially abundant proteins were assessed using STRING software with a high confidence level of 0.700.

### 4.4. IHC

Three proteins identified in the PDL by proteomic analysis were selected. Their presence was confirmed by IHC using additional histological sections from the same animals assessed by LC-MS/MS. IHC was performed on paraffin sections using an avidin-biotinylated peroxidase enzyme complex- (ABC) based kit (Vector Labs, Burlingame, CA, USA) with 3-amino-9-ethylcarbazole (AEC) chromogenic substrate (Vector Labs, Burlingame, CA, USA), as described previously [63]. The primary antibodies included were Prelp (bs13707R, Bioss, Hong Kong, China), Sec13 (ORB312990, Biorbyt, Cambridge, UK), and Sod2 (PA530604, Invitrogen). Samples were stained with 3,3′-diaminobenzidine (DAB, Dako, Glostrup, Denmark) and counterstained with Carazzi’s hematoxylin. Negative controls lacking primary antibodies were performed. Representative photomicrographs of each sample’s mesial, distal, and furcation areas per group were taken at 400x magnification using a digital camera (DP-71, Olympus, Hong Kong, China) attached to a light microscope (BX-51, Olympus). The analysis was performed by an experienced examiner blinded to the experimental groups by counting positive cells.

### 4.5. Statistical Analysis

The statistical *t*-test with a Bonferroni test correction was used (*p* < 0.05) to determine the differential protein abundances on average peak area values of the control and obese groups. The fold-change was calculated as the ratio of obese/control values. For the IHC analysis, data were tested for normality using the Shapiro-Wilk test and an intergroup comparison was performed using the t-test (Graph-Pad Prism 9 Software Inc., San Diego, CA, USA) at a significance level of 5%.

## Figures and Tables

**Table 1:** List of differentially abundant proteins related to obesity in the GO term “extracellular exosome” in the PDL of control and obese groups. Average peak area values of each group, *p*-values (*t*-test) and fold-change are shown.

Extracellular Exosome (GO:0070062)						
			Average Peak Area Values			
Protein Name	Uniprot Accession	Protein Symbol	Control	Obese	*p*-Value	Fold-Change
Prolargin	Q9EQP5	Prelp	0.00	1.20 × 10^7^	0.014 *	1.2 × 10^7^
Protein SEC13 homolog	Q5XFW8	Sec13	7.40 × 10^5^	4.60 × 10^6^	0.014 *	6.10
Superoxide dismutase	P07895	Sod2	2.60 × 10^6^	7.80 × 10^6^	0.040 *	3.00

* Significant (*p* < 0.05) difference.

## Data Availability

Not applicable.

## Supplementary Materials

The following supporting information can be downloaded at: https://www.mdpi.com/article/10.3390/ijms24021003/s1.
