# Supplementary material for: Obesity Modifies the Proteomic Profile of the Periodontal Ligament
Source: Int J Mol Sci. 2023 Jan 5;24(2):1003. doi: 10.3390/ijms24021003 (PMC9861657; doi:10.3390/ijms24021003)
Supplement: Supplementary file 1 [file ijms-24-01003-s001.zip › Figure S1 - Gene report.pdf]

Gene Report

Help and Manual

Download File

<table>
<tr><th>List Id: P35427</th><th>ribosomal protein L13A(Rpl13a)</th><th>Related Genes</th><th>Rattus norvegicus</th></tr>
<tr><td>CHROMOSOME</td><td colspan="3">1,</td></tr>
<tr><td>CYTOGENETIC_LOCATION</td><td colspan="3">1q22,</td></tr>
<tr><td>ENSEMBL_GENE_ID</td><td colspan="3">ENSRNOG00000020618,</td></tr>
<tr><td>ENTREZ_GENE_ID</td><td colspan="3">317646,</td></tr>
<tr><td>GENERIF_SUMMARY</td><td colspan="3">Data show that EF1alpha, RPL13a and YWHAZ are suitable genes for the RT-qPCR analysis and comparison of several sources of human MSC during in vitro characterization and differentiation as well as in an ex vivo animal model of global cerebral ischemia., We evaluated copy number variation (CNV) for four genes in rat strains differing in nervous system excitability. rpl13a copy number is significantly reduced in hippocampus and bone marrow in rats with a high excitability threshold and stress,</td></tr>
<tr><td>UNIPROT_ID</td><td colspan="3">A0A1W2Q602_RAT, Q5RK10_RAT, RL13A_RAT,</td></tr>
<tr><td>UP_COMMENT_FUNCTION</td><td colspan="3">Associated with ribosomes but is not required for canonical ribosome function and has extra-ribosomal functions. Component of the GAIT (gamma interferon-activated inhibitor of translation) complex which mediates interferon-gamma-induced transcript-selective translation inhibition in inflammation processes. Upon interferon-gamma activation and subsequent phosphorylation dissociates from the ribosome and assembles into the GAIT complex which binds to stem loop-containing GAIT elements in the 3'-UTR of diverse inflammatory mRNAs (such as ceruplasmin) and suppresses their translation. In the GAIT complex interacts with m7G cap-bound eIF4G at or near the eIF3-binding site and blocks the recruitment of the 43S ribosomal complex. Involved in methylation of rRNA. {ECO:0000250|UniProtKB:P40429}.,</td></tr>
<tr><td>UP_COMMENT_PTM</td><td colspan="3">Citrullinated by PADI4. {ECO:0000250|UniProtKB:P19253}., Phosphorylation at Ser-77 upon interferon-gamma treatment in macrophages involves a DAPK1-DAPK3 kinase cascade and is causing release from the ribosome, association with the GAIT complex and subsequent involvement in transcript-selective translation inhibition. {ECO:0000250|UniProtKB:P19253}.,</td></tr>
<tr><td>UP_COMMENT_SIMILARITY</td><td colspan="3">Belongs to the universal ribosomal protein uL13 family. {ECO:0000256|ARBA:ARBA00006227, ECO:0000256|RuleBase:RU003877}., Belongs to the universal ribosomal protein uL13 family. {ECO:0000256|ARBA:ARBA00006227}., Belongs to the universal ribosomal protein uL13 family. {ECO:0000305}.,</td></tr>
<tr><td>UP_COMMENT_SUBCELLULAR_LOCATION</td><td colspan="3">Cytoplasm {ECO:0000250|UniProtKB:P40429}.,</td></tr>
<tr><td>UP_COMMENT_SUBUNIT</td><td colspan="3">Component of the 60S ribosome. Component of the GAIT complex. Interacts with EIF4G1. {ECO:0000250|UniProtKB:P40429}., Component of the 60S ribosome. Component of the GAIT complex. Interacts with EIF4G1. {ECO:0000256|ARBA:ARBA00026018}.,</td></tr>
<tr><th>List Id: F1LSW7</th><th>ribosomal protein L14(Rpl14)</th><th>Related Genes</th><th>Rattus norvegicus</th></tr>
<tr><td>CHROMOSOME</td><td colspan="3">8,</td></tr>
<tr><td>CYTOGENETIC_LOCATION</td><td colspan="3">8q32,</td></tr>
<tr><td>ENSEMBL_GENE_ID</td><td colspan="3">ENSRNOG00000019007,</td></tr>
<tr><td>ENTREZ_GENE_ID</td><td colspan="3">65043,</td></tr>
<tr><td>UNIPROT_ID</td><td colspan="3">A0A0G2K570_RAT, A0A8I5ZPM1_RAT, B5DEM5_RAT, F1LSW7_RAT, RL14_RAT,</td></tr>
<tr><td>UP_COMMENT_FUNCTION</td><td colspan="3">Component of the large ribosomal subunit. The ribosome is a large ribonucleoprotein complex responsible for the synthesis of proteins in the cell. {ECO:0000250|UniProtKB:P50914}.,</td></tr>
<tr><td>UP_COMMENT_SIMILARITY</td><td colspan="3">Belongs to the eukaryotic ribosomal protein eL14 family. {ECO:0000256|ARBA:ARBA00006592}., Belongs to the eukaryotic ribosomal protein eL14 family. {ECO:0000305}.,</td></tr>
<tr><td>UP_COMMENT_SUBCELLULAR_LOCATION</td><td colspan="3">Cytoplasm {ECO:0000250|UniProtKB:P50914}.,</td></tr>
<tr><td>UP_COMMENT_SUBUNIT</td><td colspan="3">Component of the large ribosomal subunit. {ECO:0000250|UniProtKB:P50914}.,</td></tr>
</table>

<table>
<tr><th>List Id: Q0QEW8</th><th>ribosomal protein L18(Rpl18)</th><th>Related Genes</th><th>Rattus norvegicus</th></tr>
<tr><td>CHROMOSOME</td><td colspan="3">1,</td></tr>
<tr><td>CYTOGENETIC_LOCATION</td><td colspan="3">1q22,</td></tr>
<tr><td>ENSEMBL_GENE_ID</td><td colspan="3">ENSRNOG00000021035,</td></tr>
<tr><td>ENTREZ_GENE_ID</td><td colspan="3">81766,</td></tr>
<tr><td>UNIPROT_ID</td><td colspan="3">A0A0H2UHS7_RAT, A0A8I5YC25_RAT, A0A8I6A4M9_RAT, A0A8I6B2T7_RAT, A0A8I6GD73_RAT, Q0QEW8_RAT, RL18_RAT,</td></tr>
<tr><td>UP_COMMENT_FUNCTION</td><td colspan="3">Component of the large ribosomal subunit. The ribosome is a large ribonucleoprotein complex responsible for the synthesis of proteins in the cell. {ECO:0000250|UniProtKB:Q07020}., Component of the large ribosomal subunit. {ECO:0000256|ARBA:ARBA00002814}.,</td></tr>
<tr><td>UP_COMMENT_SIMILARITY</td><td colspan="3">Belongs to the eukaryotic ribosomal protein eL18 family. {ECO:0000256|ARBA:ARBA00006815}., Belongs to the eukaryotic ribosomal protein eL18 family. {ECO:0000305}.,</td></tr>
<tr><td>UP_COMMENT_SUBCELLULAR_LOCATION</td><td colspan="3">Cytoplasm, cytosol {ECO:0000250|UniProtKB:Q07020}. Cytoplasm {ECO:0000250|UniProtKB:Q07020}. Rough endoplasmic reticulum {ECO:0000250|UniProtKB:Q95342}. Note=Detected on cytosolic polysomes (By similarity). Detected in ribosomes that are associated with the rough endoplasmic reticulum (By similarity). {ECO:0000250|UniProtKB:Q07020, ECO:0000250|UniProtKB:Q95342}., Endoplasmic reticulum {ECO:0000256|ARBA:ARBA00004240}. Rough endoplasmic reticulum {ECO:0000256|ARBA:ARBA00004427}.,</td></tr>
<tr><td>UP_COMMENT_SUBUNIT</td><td colspan="3">Component of the large ribosomal subunit. {ECO:0000250|UniProtKB:Q07020}., Component of the large ribosomal subunit. {ECO:0000256|ARBA:ARBA00011133}.,</td></tr>
<tr><th>List Id: Q6PDV8</th><th>ribosomal protein L22, pseudogene 2(Rpl22-ps2)</th><th>Related Genes</th><th>Rattus norvegicus</th></tr>
<tr><td>CHROMOSOME</td><td colspan="3">4,</td></tr>
<tr><td>CYTOGENETIC_LOCATION</td><td colspan="3">4q34,</td></tr>
<tr><td>ENSEMBL_GENE_ID</td><td colspan="3">ENSRNOG00000011104, ENSRNOG00000028747,</td></tr>
<tr><td>ENTREZ_GENE_ID</td><td colspan="3">100360057,</td></tr>
<tr><td>UNIPROT_ID</td><td colspan="3">A0A8J8XZ98_RAT, Q6PDV8_RAT,</td></tr>
<tr><td>UP_COMMENT_SIMILARITY</td><td colspan="3">Belongs to the eukaryotic ribosomal protein eL22 family. {ECO:0000256|ARBA:ARBA00007817}.,</td></tr>
<tr><th>List Id: F1M013</th><th>ribosomal protein L7A(Rpl7a)</th><th>Related Genes</th><th>Rattus norvegicus</th></tr>
<tr><td>CHROMOSOME</td><td colspan="3">3,</td></tr>
<tr><td>CYTOGENETIC_LOCATION</td><td colspan="3">3p13,</td></tr>
<tr><td>ENSEMBL_GENE_ID</td><td colspan="3">ENSRNOG00000047737, ENSRNOG00000070974,</td></tr>
<tr><td>ENTREZ_GENE_ID</td><td colspan="3">296596,</td></tr>
<tr><td>UNIPROT_ID</td><td colspan="3">A0A8J8YIF6_RAT, B0K021_RAT, F1M013_RAT, RL7A_RAT,</td></tr>
<tr><td>UP_COMMENT_FUNCTION</td><td colspan="3">Component of the large ribosomal subunit. The ribosome is a large ribonucleoprotein complex responsible for the synthesis of proteins in the cell. {ECO:0000250|UniProtKB:P62424}., Component of the ribosome. {ECO:0000256|RuleBase:RU367042}.,</td></tr>
<tr><td>UP_COMMENT_SIMILARITY</td><td colspan="3">Belongs to the eukaryotic ribosomal protein eL8 family. {ECO:0000256|ARBA:ARBA00007337, ECO:0000256|RuleBase:RU367042}., Belongs to the eukaryotic ribosomal protein eL8 family. {ECO:0000305}.,</td></tr>
<tr><td>UP_COMMENT_SUBCELLULAR_LOCATION</td><td colspan="3">Cytoplasm {ECO:0000250|UniProtKB:P62424}.,</td></tr>
<tr><td>UP_COMMENT_SUBUNIT</td><td colspan="3">Component of the large ribosomal subunit (By similarity). Interacts with CRY1 (By similarity). Interacts with DICER1, AGO2, TARBP2, MOV10 and EIF6; they form a large RNA-induced silencing complex (RISC) (By similarity). {ECO:0000250|UniProtKB:P12970, ECO:0000250|UniProtKB:P62424}.,</td></tr>
<tr><th>List Id: P62250</th><th>ribosomal protein S16(Rps16)</th><th>Related Genes</th><th>Rattus norvegicus</th></tr>
<tr><td>CHROMOSOME</td><td colspan="3">1,</td></tr>
<tr><td>CYTOGENETIC_LOCATION</td><td colspan="3">1q21,</td></tr>
<tr><td>ENSEMBL_GENE_ID</td><td colspan="3">ENSRNOG00000019578,</td></tr>
<tr><td>ENTREZ_GENE_ID</td><td colspan="3">140655,</td></tr>
<tr><td>UNIPROT_ID</td><td colspan="3">A0A1W2Q631_RAT, A0A8L2QED1_RAT, B0K038_RAT, Q5XFV9_RAT, Q6P3E1_RAT, RS16_RAT,</td></tr>
<tr><td>UP_COMMENT_SIMILARITY</td><td colspan="3">Belongs to the universal ribosomal protein uS9 family. {ECO:0000256|ARBA:ARBA00005251, ECO:0000256|RuleBase:RU003815}., Belongs to the universal ribosomal protein uS9 family. {ECO:0000305}.,</td></tr>
<tr><th>List Id: M0RA26</th><th>ribosomal protein S27-like(LOC100362987)</th><th>Related Genes</th><th>Rattus norvegicus</th></tr>
<tr><td>CHROMOSOME</td><td colspan="3">9,</td></tr>
<tr><td>CYTOGENETIC_LOCATION</td><td colspan="3">9q12,</td></tr>
<tr><td>ENSEMBL_GENE_ID</td><td colspan="3">ENSRNOG00000021159,</td></tr>
<tr><td>ENTREZ_GENE_ID</td><td colspan="3">100362987,</td></tr>
<tr><td>UNIPROT_ID</td><td colspan="3">M0RA26_RAT,</td></tr>
<tr><td>UP_COMMENT_COFACTOR</td><td colspan="3">Name=Zn(2+); Xref=ChEBI:CHEBI:29105; Evidence={ECO:0000256|ARBA:ARBA00001947};,</td></tr>
<tr><td>UP_COMMENT_SIMILARITY</td><td colspan="3">Belongs to the eukaryotic ribosomal protein eS27 family. {ECO:0000256|ARBA:ARBA00010919}.,</td></tr>
<tr><th>List Id: P62275</th><th>ribosomal protein S29(Rps29)</th><th>Related Genes</th><th>Rattus norvegicus</th></tr>
<tr><td>CHROMOSOME</td><td colspan="3">6,</td></tr>
<tr><td>CYTOGENETIC_LOCATION</td><td colspan="3">6q24,</td></tr>
<tr><td>ENSEMBL_GENE_ID</td><td colspan="3">ENSRNOG00000004196,</td></tr>
<tr><td>ENTREZ_GENE_ID</td><td colspan="3">25348,</td></tr>
<tr><td>UNIPROT_ID</td><td colspan="3">A0A8I6GMA6_RAT, RS29_RAT,</td></tr>
<tr><td>UP_COMMENT_COFACTOR</td><td colspan="3">Name=Zn(2+); Xref=ChEBI:CHEBI:29105; Evidence={ECO:0000250|UniProtKB:P62273}; Note=Binds 1 zinc ion per subunit. {ECO:0000250|UniProtKB:P62273};, Name=Zn(2+); Xref=ChEBI:CHEBI:29105; Evidence={ECO:0000256|ARBA:ARBA00001947};,</td></tr>
<tr><td>UP_COMMENT_SIMILARITY</td><td colspan="3">Belongs to the universal ribosomal protein uS14 family. {ECO:0000256|ARBA:ARBA00009083}., Belongs to the universal ribosomal protein uS14 family. {ECO:0000305}.,</td></tr>
<tr><td>UP_COMMENT_SUBCELLULAR_LOCATION</td><td colspan="3">Cytoplasm, cytosol {ECO:0000250|UniProtKB:P62273}. Cytoplasm {ECO:0000250|UniProtKB:P62273}. Rough endoplasmic reticulum {ECO:0000250|UniProtKB:Q6QAP6}. Note=Detected on cytosolic polysomes (By similarity). Detected in ribosomes that are associated with the rough endoplasmic reticulum (By similarity). {ECO:0000250|UniProtKB:P62273, ECO:0000250|UniProtKB:Q6QAP6}.,</td></tr>
<tr><td>UP_COMMENT_SUBUNIT</td><td colspan="3">Component of the 40S small ribosomal subunit. {ECO:0000250|UniProtKB:Q6QAP6}., Component of the 40S small ribosomal subunit. {ECO:0000256|ARBA:ARBA00011542}.,</td></tr>
</table>

<table>
<tr><th>List Id: P62909</th><th>ribosomal protein S3(Rps3)</th><th>Related Genes</th><th>Rattus norvegicus</th></tr>
<tr><td>CHROMOSOME</td><td colspan="3">1,</td></tr>
<tr><td>CYTOGENETIC_LOCATION</td><td colspan="3">1q32,</td></tr>
<tr><td>ENSEMBL_GENE_ID</td><td colspan="3">ENSRNOG00000017418,</td></tr>
<tr><td>ENTREZ_GENE_ID</td><td colspan="3">140654,</td></tr>
<tr><td>GENERIF_SUMMARY</td><td colspan="3">Findings define an extraribosomal role of RPS3 as a molecular switch that accommodates apoptotic induction to DNA repair through Akt-mediated phosphorylation.,</td></tr>
<tr><td>UNIPROT_ID</td><td colspan="3">A0A8I5ZK30_RAT, A0A8I6A8D6_RAT, A0A8L2QCG7_RAT, RS3_RAT,</td></tr>
<tr><td>UP_COMMENT_CATALYTIC_ACTIVITY</td><td colspan="3">Reaction=2'-deoxyribonucleotide-(2'-deoxyribose 5'-phosphate)-2'- deoxyribonucleotide-DNA = a 3'-end 2'-deoxyribonucleotide-(2,3- dehydro-2,3-deoxyribose 5'-phosphate)-DNA + a 5'-end 5'-monophospho- 2'-deoxyribonucleoside-DNA + H(+); Xref=Rhea:RHEA:66592, Rhea:RHEA- COMP:13180, Rhea:RHEA-COMP:16897, Rhea:RHEA-COMP:17067, ChEBI:CHEBI:15378, ChEBI:CHEBI:136412, ChEBI:CHEBI:157695, ChEBI:CHEBI:167181; EC=4.2.99.18; Evidence={ECO:0000250|UniProtKB:P23396};, Reaction=2'-deoxyribonucleotide-(2'-deoxyribose 5'-phosphate)-2'- deoxyribonucleotide-DNA = a 3'-end 2'-deoxyribonucleotide-(2,3- dehydro-2,3-deoxyribose 5'-phosphate)-DNA + a 5'-end 5'-monophospho- 2'-deoxyribonucleoside-DNA + H(+); Xref=Rhea:RHEA:66592, Rhea:RHEA- COMP:13180, Rhea:RHEA-COMP:16897, Rhea:RHEA-COMP:17067, ChEBI:CHEBI:15378, ChEBI:CHEBI:136412, ChEBI:CHEBI:157695, ChEBI:CHEBI:167181; EC=4.2.99.18; Evidence={ECO:0000256|ARBA:ARBA00024490};,</td></tr>
<tr><td>UP_COMMENT_FUNCTION</td><td colspan="3">Involved in translation as a component of the 40S small ribosomal subunit (By similarity). Has endonuclease activity and plays a role in repair of damaged DNA (PubMed:7775413). Cleaves phosphodiester bonds of DNAs containing altered bases with broad specificity and cleaves supercoiled DNA more efficiently than relaxed DNA (By similarity). Displays high binding affinity for 7,8-dihydro-8- oxoguanine (8-oxoG), a common DNA lesion caused by reactive oxygen species (ROS) (By similarity). Has also been shown to bind with similar affinity to intact and damaged DNA (By similarity). Stimulates the N- glycosylase activity of the base excision protein OGG1 (By similarity). Enhances the uracil excision activity of UNG1 (By similarity). Also stimulates the cleavage of the phosphodiester backbone by APEX1 (By similarity). When located in the mitochondrion, reduces cellular ROS levels and mitochondrial DNA damage. Has also been shown to negatively regulate DNA repair in cells exposed to hydrogen peroxide (By similarity). Plays a role in regulating transcription as part of the NF-kappa-B p65-p50 complex where it binds to the RELA/p65 subunit, enhances binding of the complex to DNA and promotes transcription of target genes (By similarity). Represses its own translation by binding to its cognate mRNA (By similarity). Binds to and protects TP53/p53 from MDM2-mediated ubiquitination (By similarity). Involved in spindle formation and chromosome movement during mitosis by regulating microtubule polymerization (By similarity). Involved in induction of apoptosis through its role in activation of CASP8 (By similarity). Induces neuronal apoptosis by interacting with the E2F1 transcription factor and acting synergistically with it to up-regulate pro-apoptotic proteins BCL2L11/BIM and HRK/Dp5 (By similarity). Interacts with TRADD following exposure to UV radiation and induces apoptosis by caspase-dependent JNK activation (By similarity). {ECO:0000250|UniProtKB:P23396, ECO:0000269|PubMed:7775413}.,</td></tr>
<tr><td>UP_COMMENT_PTM</td><td colspan="3">Methylation by PRMT1 is required for import into the nucleolus and for ribosome assembly. {ECO:0000250|UniProtKB:P23396}., Phosphorylation at Thr-221 by CDK1 occurs mainly in G2/M phase. Phosphorylation by PRKCD occurs on a non-ribosomal-associated form which results in translocation of RPS3 to the nucleus and enhances its endonuclease activity. Phosphorylated on Ser-209 by IKKB in response to activation of the NF-kappa-B p65-p50 complex which enhances the association of RPS3 with importin-alpha and mediates the nuclear translocation of RPS3. Phosphorylation by MAPK is required for translocation to the nucleus following exposure of cells to DNA damaging agents such as hydrogen peroxide. Phosphorylation by PKB/AKT mediates RPS3 nuclear translocation, enhances RPS3 endonuclease activity and suppresses RPS3-induced neuronal apoptosis. {ECO:0000250|UniProtKB:P23396}., Sumoylation by SUMO1 enhances protein stability through increased resistance to proteolysis. Sumoylation occurs at one or more of the three consensus sites, Lys-18, Lys-214 and Lys-230. {ECO:0000250|UniProtKB:P23396}., Ubiquitinated. This is prevented by interaction with HSP90 which stabilizes the protein. Monoubiquitinated at Lys-214 by ZNF598 when a ribosome has stalled during translation of poly(A) sequences, leading to preclude synthesis of a long poly-lysine tail and initiate the ribosome quality control (RQC) pathway to degrade the potentially detrimental aberrant nascent polypeptide. {ECO:0000250|UniProtKB:P23396}., Ufmylated by UFL1. {ECO:0000250|UniProtKB:P62908}.,</td></tr>
<tr><td>UP_COMMENT_SIMILARITY</td><td colspan="3">Belongs to the universal ribosomal protein uS3 family. {ECO:0000256|ARBA:ARBA00010761}., Belongs to the universal ribosomal protein uS3 family. {ECO:0000305}.,</td></tr>
<tr><td>UP_COMMENT_SUBCELLULAR_LOCATION</td><td colspan="3">Cytoplasm {ECO:0000250|UniProtKB:P23396}. Nucleus {ECO:0000250|UniProtKB:P23396}. Nucleus, nucleolus {ECO:0000250|UniProtKB:P23396}. Mitochondrion inner membrane {ECO:0000250|UniProtKB:P23396}; Peripheral membrane protein {ECO:0000250|UniProtKB:P23396}. Cytoplasm, cytoskeleton, spindle {ECO:0000250|UniProtKB:P23396}. Note=In normal cells, located mainly in the cytoplasm with small amounts in the nucleus but translocates to the nucleus in cells undergoing apoptosis. Nuclear translocation is induced by DNA damaging agents such as hydrogen peroxide. Accumulates in the mitochondrion in response to increased ROS levels. Localizes to the spindle during mitosis. Localized in cytoplasmic mRNP granules containing untranslated mRNAs. {ECO:0000250|UniProtKB:P23396, ECO:0000250|UniProtKB:P62908}., Cytoplasm, cytoskeleton, spindle {ECO:0000256|ARBA:ARBA00004186}. Membrane {ECO:0000256|ARBA:ARBA00004170}; Peripheral membrane protein {ECO:0000256|ARBA:ARBA00004170}. Mitochondrion inner membrane {ECO:0000256|ARBA:ARBA00004637}; Peripheral membrane protein {ECO:0000256|ARBA:ARBA00004637}. Nucleus, nucleolus {ECO:0000256|ARBA:ARBA00004604}., Membrane {ECO:0000256|ARBA:ARBA00004170}; Peripheral membrane protein {ECO:0000256|ARBA:ARBA00004170}. Mitochondrion inner membrane {ECO:0000256|ARBA:ARBA00004637}; Peripheral membrane protein {ECO:0000256|ARBA:ARBA00004637}.,</td></tr>
<tr><td>UP_COMMENT_SUBUNIT</td><td colspan="3">Component of the 40S small ribosomal subunit. Identified in a IGF2BP1-dependent mRNP granule complex containing untranslated mRNAs. Interacts with HNRPD. Interacts with PRMT1; the interaction methylates RPS3. Interacts with SUMO1; the interaction sumoylates RPS3. Interacts with UBC9. Interacts with CDK1; the interaction phosphorylates RPS3. Interacts with PRKCD; the interaction phosphorylates RPS3. Interacts with PKB/AKT; the interaction phosphorylates RPS3. Interacts with E2F1; the interaction occurs in the absence of nerve growth factor and increases transcription of pro-apoptotic proteins BCL2L11/BIM and HRK/Dp5. Interacts with the base excision repair proteins APEX1 and OGG1; interaction with OGG1 increases OGG1 N-glycosylase activity. Interacts with UNG; the interaction increases the uracil excision activity of UNG1. Interacts with HSP90; the interaction prevents the ubiquitination and proteasome-dependent degradation of RPS3 and is suppressed by increased ROS levels. Interacts with TOM70; the interaction promotes translocation of RPS3 to the mitochondrion. Interacts (via N-terminus) with RELA (via N-terminus); the interaction enhances the DNA-binding activity of the NF-kappa-B p65-p50 complex. Interacts with NFKBIA; the interaction is direct and may bridge the interaction between RPS3 and RELA. Interacts with IKKB; the interaction phosphorylates RPS3 and enhances its translocation to the nucleus. Interacts (via KH domain) with MDM2 and TP53. Interacts with TRADD. Interacts with CRY1. {ECO:0000250|UniProtKB:P23396, ECO:0000250|UniProtKB:P62908}.,</td></tr>
<tr><th>List Id: P62083</th><th>ribosomal protein S7(Rps7)</th><th>Related Genes</th><th>Rattus norvegicus</th></tr>
<tr><td>CHROMOSOME</td><td colspan="3">6,</td></tr>
<tr><td>CYTOGENETIC_LOCATION</td><td colspan="3">6q16,</td></tr>
<tr><td>ENSEMBL_GENE_ID</td><td colspan="3">ENSRNOG00000008373,</td></tr>
<tr><td>ENTREZ_GENE_ID</td><td colspan="3">29258,</td></tr>
<tr><td>UNIPROT_ID</td><td colspan="3">A0A8I6ABK7_RAT, A0A8L2Q5A3_RAT, A0A8L2RB77_RAT, B5DEL9_RAT, RS7_RAT,</td></tr>
<tr><td>UP_COMMENT_FUNCTION</td><td colspan="3">Required for rRNA maturation. {ECO:0000250|UniProtKB:P62081}., Required for rRNA maturation. {ECO:0000256|ARBA:ARBA00003567}.,</td></tr>
<tr><td>UP_COMMENT_PTM</td><td colspan="3">Phosphorylated by NEK6. {ECO:0000250|UniProtKB:P62081}., Ubiquitinated. Deubiquitinated by DESI2, leading to its stabilization. {ECO:0000250|UniProtKB:P62081}.,</td></tr>
<tr><td>UP_COMMENT_SIMILARITY</td><td colspan="3">Belongs to the eukaryotic ribosomal protein eS7 family. {ECO:0000256|ARBA:ARBA00007820, ECO:0000256|RuleBase:RU364105}., Belongs to the eukaryotic ribosomal protein eS7 family. {ECO:0000305}.,</td></tr>
<tr><td>UP_COMMENT_SUBCELLULAR_LOCATION</td><td colspan="3">Cytoplasm, cytoskeleton, microtubule organizing center, centrosome {ECO:0000250|UniProtKB:P62081}. Cytoplasm {ECO:0000250|UniProtKB:P62081}. Nucleus {ECO:0000250|UniProtKB:P62081}. Note=Although RPS7 is functional within the cytoplasm, the assembly of ribosomal subunits occurs in the nucleus. RPS7 nuclear import is mediated by IPO5/RanBP5, IPO7/RanBP7, KPNB1/importin-beta or TPNO1/Trn (By similarity). Colocalizes with NEK6 in the centrosome (By similarity). {ECO:0000250|UniProtKB:P62081}.,</td></tr>
<tr><td>UP_COMMENT_SUBUNIT</td><td colspan="3">Binds IPO9 with high affinity (By similarity). Interacts with NEK6 (By similarity). Interacts with DESI2 (By similarity). Interacts with IPO5, IPO7 and KPNB1; these interactions may be involved in RPS7 nuclear import for the assembly of ribosomal subunits (By similarity). {ECO:0000250|UniProtKB:P62081}.,</td></tr>
<tr><th>List Id: A0A0H2UHU0</th><th>ribosomal protein s25(Rps25)</th><th>Related Genes</th><th>Rattus norvegicus</th></tr>
<tr><td>CHROMOSOME</td><td colspan="3">8,</td></tr>
<tr><td>CYTOGENETIC_LOCATION</td><td colspan="3">8q22,</td></tr>
<tr><td>ENSEMBL_GENE_ID</td><td colspan="3">ENSRNOG00000027503,</td></tr>
<tr><td>ENTREZ_GENE_ID</td><td colspan="3">122799,</td></tr>
<tr><td>UNIPROT_ID</td><td colspan="3">A0A0H2UHU0_RAT, A0A8I5ZLX3_RAT, A0A8I6GLY7_RAT, RS25_RAT,</td></tr>
<tr><td>UP_COMMENT_SIMILARITY</td><td colspan="3">Belongs to the eukaryotic ribosomal protein eS25 family. {ECO:0000256|ARBA:ARBA00009106, ECO:0000256|RuleBase:RU366057}., Belongs to the eukaryotic ribosomal protein eS25 family. {ECO:0000305}.,</td></tr>
</table>
