# Supplementary material for: Obesity Modifies the Proteomic Profile of the Periodontal Ligament
Source: Int J Mol Sci. 2023 Jan 5;24(2):1003. doi: 10.3390/ijms24021003 (PMC9861657; doi:10.3390/ijms24021003)
Supplement: Supplementary file 1 [file ijms-24-01003-s001.zip › Table S1.pdf]

Table S1 - List of differentially abundant proteins in PDL of the control and obese groups. Average peak area values of each group, p-values (*t*-test), and fold-change (obese / control) are shown.

Accession	Description	Protein Symbol	Peak area										*t*-test	Fold-Change
			Control 1	Control 2	Control 3	Control Average	Obese 1	Obese 2	Obese 3	Obese 4	Obese 5	Obese Average		
**Up-regulated in Obesity**														
Q9EQP5	Prolargin	Prelp	0.0E0	0.0E0	0.0E0	**0.0E0**	5.4E6	1.3E7	1.8E7	1.5E7	6.3E6	**1.2E7**	0.014	**1.2E7**
Q4KLF8	Actin-related protein 2/3 complex subunit 5	Arpc5	0.0E0	0.0E0	0.0E0	**0.0E0**	1.0E7	9.1E6	0.0E0	7.4E6	5.4E6	**6.4E6**	0.036	**6.4E6**
I6L9G6	RCG31562, isoform CRA_c	I6l9g6	0.0E0	0.0E0	0.0E0	**0.0E0**	4.0E6	3.6E6	1.7E6	2.4E6	0.0E0	**2.4E6**	0.049	**2.4E6**
Q9EPC6	Profilin-2	Prof2	0.0E0	0.0E0	0.0E0	**0.0E0**	3.1E6	3.0E6	3.4E6	0.0E0	1.3E6	**2.2E6**	0.046	**2.2E6**
A3FM27	Chloride intracellular channel protein	A3fm27	0.0E0	0.0E0	0.0E0	**0.0E0**	1.4E6	1.5E6	1.4E6	2.6E6	0.0E0	**1.4E6**	0.043	**1.4E6**
Q5XFW8	Protein SEC13 homolog	Sec13	0.0E0	2.2E6	0.0E0	**7.4E5**	2.7E6	5.9E6	5.0E6	6.2E6	3.0E6	**4.6E6**	0.014	**6.1**
O35796	Complement component 1 Q subcomponent-binding protein, mitochondrial	C1qbp	0.0E0	2.9E6	0.0E0	**9.5E5**	3.4E6	4.0E6	2.5E6	6.1E6	4.7E6	**4.1E6**	0.026	**4.3**
P07895	Superoxide dismutase	Sod2	0.0E0	3.5E6	4.3E6	**2.6E6**	4.9E6	4.7E6	1.1E7	8.6E6	9.4E6	**7.8E6**	0.040	**3.0**
P17764	Acetyl-CoA acetyltransferase, mitochondrial	Thil	0.0E0	1.6E6	3.1E6	**1.6E6**	4.0E6	4.8E6	3.8E6	6.1E6	4.5E6	**4.6E6**	0.011	**3.0**
P16975	SPARC	Sprc	1.1E7	1.5E7	8.2E6	**1.1E7**	1.5E7	2.6E7	1.8E7	1.9E7	1.7E7	**1.9E7**	0.042	**1.7**
**Down-regulated in Obesity**														
A0A0G2K0W9	Proteasome subunit alpha type	A0a0g2k0w9	4.4E6	4.8E6	3.4E6	**4.2E6**	3.3E6	2.9E6	3.2E6	3.6E6	2.2E6	**3.0E6**	0.037	**-1.4**
A0A0U1RRV7	RCG61099, isoform CRA_b	A0a0u1rrv7	1.3E7	1.3E7	1.6E7	**1.4E7**	8.5E6	8.1E6	1.2E7	1.1E7	8.0E6	**9.5E6**	0.018	**-1.5**
M0RA26	Ribosomal protein S27-like	M0ra26	8.3E6	1.1E7	8.2E6	**9.2E6**	8.2E6	7.0E6	5.1E6	6.4E6	4.2E6	**6.2E6**	0.044	**-1.5**
D3ZWE0	Histone H2A	D3zwe0	6.5E8	6.5E8	6.1E8	**6.4E8**	4.5E8	4.1E8	4.9E8	5.4E8	2.4E8	**4.3E8**	0.022	**-1.5**
P61805	Dolichyl-diphosphooligosaccharide--protein glycosyltransferase subunit DAD1	Dad1	9.1E6	7.9E6	5.4E6	**7.5E6**	5.2E6	4.6E6	6.1E6	5.0E6	3.3E6	**4.9E6**	0.039	**-1.5**
A0A0G2K757	Dolichyl-diphosphooligosaccharide--protein glycosyltransferase subunit 2	A0a0g2k757	2.7E7	2.4E7	1.7E7	**2.2E7**	1.5E7	1.5E7	1.3E7	2.0E7	9.1E6	**1.4E7**	0.049	**-1.5**

Q794E4	Heterogeneous nuclear ribonucleoprotein F	Hnrpf	1.8E7	1.9E7	1.5E7	**1.7E7**	1.4E7	9.5E6	1.1E7	9.9E6	1.1E7	**1.1E7**	0.002	**-1.6**
Q68FP1	Gelsolin	Gels	6.9E7	6.7E7	4.3E7	**6.0E7**	4.2E7	4.4E7	3.3E7	4.2E7	2.5E7	**3.7E7**	0.028	**-1.6**
F1M779	Clathrin heavy chain	F1m779	4.8E7	4.5E7	2.7E7	**4.0E7**	2.6E7	2.7E7	1.9E7	3.2E7	2.0E7	**2.5E7**	0.041	**-1.6**
Q4V7D1	Signal sequence receptor, alpha	Q4v7d1	2.1E7	2.5E7	1.7E7	**2.1E7**	1.8E7	1.1E7	1.2E7	1.4E7	7.8E6	**1.3E7**	0.026	**-1.7**
Q6AXS5	Plasminogen activator inhibitor 1 RNA-binding protein	Pairb	3.8E6	4.9E6	3.6E6	**4.1E6**	2.5E6	2.4E6	2.1E6	3.9E6	1.3E6	**2.4E6**	0.038	**-1.7**
P62909	40S ribosomal protein S3	Rs3	5.0E7	4.3E7	3.4E7	**4.2E7**	2.8E7	2.8E7	2.4E7	2.7E7	1.5E7	**2.4E7**	0.009	**-1.7**
P35427	60S ribosomal protein L13a	Rl13a	1.9E7	2.3E7	1.4E7	**1.9E7**	1.1E7	1.2E7	9.5E6	1.3E7	7.3E6	**1.1E7**	0.019	**-1.7**
F1LSW7	60S ribosomal protein L14	F1lsw7	2.9E7	2.8E7	1.9E7	**2.5E7**	1.7E7	1.4E7	1.3E7	2.0E7	7.7E6	**1.4E7**	0.025	**-1.8**
P31000	Vimentin	Vime	5.2E8	6.5E8	4.3E8	**5.3E8**	2.8E8	2.8E8	2.6E8	4.6E8	2.3E8	**3.0E8**	0.019	**-1.8**
A0A0G2JUA5	AHNAK nucleoprotein	A0a0g2jua5	2.0E8	2.0E8	9.5E7	**1.6E8**	1.1E8	8.1E7	8.6E7	1.2E8	6.7E7	**9.3E7**	0.047	**-1.8**
Q6AY09	Heterogeneous nuclear ribonucleoprotein H2	Hnrh2	2.1E7	2.7E7	2.0E7	**2.3E7**	1.4E7	1.5E7	1.7E7	1.1E7	6.9E6	**1.3E7**	0.013	**-1.8**
Q63030	Rat alpha-smooth muscle actin mRNA (Fragment)	Q63030	1.6E9	2.0E9	1.0E9	**1.5E9**	1.1E9	8.2E8	7.5E8	9.9E8	5.4E8	**8.4E8**	0.030	**-1.8**
Q5PQK2	FUS RNA-binding protein	Q5pqk2	6.9E6	8.2E6	5.0E6	**6.7E6**	4.2E6	3.3E6	3.9E6	4.2E6	2.9E6	**3.7E6**	0.008	**-1.8**
Q66H80	Coatomer subunit delta	Copd	8.2E6	8.1E6	4.7E6	**7.0E6**	3.6E6	4.3E6	3.5E6	5.3E6	2.6E6	**3.9E6**	0.022	**-1.8**
Q6PDU1	Serine/arginine-rich splicing factor 2	Srsf2	1.6E7	1.5E7	1.2E7	**1.4E7**	8.3E6	8.3E6	6.2E6	1.1E7	4.9E6	**7.8E6**	0.009	**-1.8**
A0A0G2JSH9	Peroxiredoxin-2	A0a0g2jsh9	3.3E7	3.5E7	1.8E7	**2.9E7**	1.6E7	1.2E7	1.7E7	2.4E7	9.7E6	**1.6E7**	0.040	**-1.8**
Q9EPB1	Dipeptidyl peptidase 2	Dpp2	3.4E7	3.1E7	1.8E7	**2.8E7**	1.8E7	2.1E7	1.4E7	1.7E7	5.0E6	**1.5E7**	0.048	**-1.8**
A0A0G2JTG7	Heterogeneous nuclear ribonucleoprotein H	A0a0g2jtg7	2.4E7	3.1E7	1.7E7	**2.4E7**	1.5E7	1.3E7	1.7E7	1.2E7	6.9E6	**1.3E7**	0.027	**-1.8**
F1LNF1	Heterogeneous nuclear ribonucleoproteins A2/B1	F1lnf1	8.0E7	7.7E7	5.3E7	**7.0E7**	3.3E7	3.6E7	3.6E7	6.4E7	2.0E7	**3.8E7**	0.029	**-1.9**
P29975	Aquaporin-1	Aqp1	1.6E7	2.2E7	9.9E6	**1.6E7**	7.5E6	7.9E6	9.5E6	1.2E7	5.2E6	**8.5E6**	0.049	**-1.9**
F1M7X3	Cadherin 13	F1m7x3	1.2E7	1.2E7	9.5E6	**1.1E7**	8.1E6	4.2E6	6.7E6	5.7E6	4.5E6	**5.9E6**	0.004	**-1.9**
A0A0H2UHT6	Ribosomal protein S18	A0a0h2uht6	4.2E7	3.9E7	2.7E7	**3.6E7**	1.7E7	2.0E7	1.4E7	3.0E7	1.3E7	**1.9E7**	0.018	**-1.9**
Q3KRF2	High density lipoprotein binding protein (Vigilin)	Q3krf2	1.8E7	1.8E7	1.1E7	**1.6E7**	7.1E6	8.2E6	7.1E6	1.3E7	5.4E6	**8.2E6**	0.026	**-1.9**

Q6S3A0	Plectin	Q6s3a0	5.1E7	5.0E7	3.0E7	**4.4E7**	2.8E7	2.2E7	1.7E7	3.0E7	1.6E7	**2.3E7**	0.016	**-1.9**
P30427	Plectin	Plec	5.1E7	5.0E7	3.0E7	**4.4E7**	2.8E7	2.2E7	1.7E7	3.0E7	1.6E7	**2.3E7**	0.016	**-1.9**
Q68FR9	Elongation factor 1-delta	Ef1d	1.6E7	2.4E7	1.8E7	**1.9E7**	1.5E7	8.3E6	8.6E6	1.3E7	5.9E6	**1.0E7**	0.014	**-1.9**
P62250	40S ribosomal protein S16	Rs16	2.9E7	2.7E7	1.2E7	**2.3E7**	1.5E7	1.2E7	1.2E7	1.2E7	6.5E6	**1.2E7**	0.049	**-1.9**
Q0QEW8	Ribosomal protein L18 (Fragment)	Q0qew8	4.2E7	3.8E7	1.9E7	**3.3E7**	1.7E7	2.1E7	1.8E7	2.1E7	8.9E6	**1.7E7**	0.038	**-1.9**
A0A0G2KA35	Uncharacterized protein	A0a0g2ka35	7.2E7	7.6E7	4.9E7	**6.6E7**	2.8E7	3.1E7	3.8E7	6.0E7	1.3E7	**3.4E7**	0.038	**-1.9**
D4A6W6	Similar to 60S ribosomal protein L8	D4a6w6	3.0E7	3.0E7	1.6E7	**2.6E7**	1.3E7	1.3E7	1.1E7	2.3E7	5.9E6	**1.3E7**	0.044	**-2.0**
P05964	Protein S100-A6	S10a6	1.4E8	1.6E8	8.3E7	**1.3E8**	6.7E7	6.0E7	6.2E7	9.0E7	4.6E7	**6.5E7**	0.020	**-2.0**
G3V8L3	Lamin A, isoform CRA_b	G3v8l3	3.6E8	4.1E8	2.1E8	**3.3E8**	1.5E8	1.5E8	1.6E8	2.5E8	1.2E8	**1.7E8**	0.024	**-2.0**
P63018	Heat shock cognate 71 kDa protein	Hsp7c	1.4E8	1.6E8	6.8E7	**1.2E8**	4.9E7	5.4E7	6.5E7	9.2E7	3.9E7	**6.0E7**	0.040	**-2.0**
A0A0G2JY31	Alpha-1-antiproteinase	A0a0g2jy31	6.5E7	5.6E7	3.1E7	**5.1E7**	2.8E7	2.2E7	2.6E7	4.1E7	9.7E6	**2.5E7**	0.046	**-2.0**
B2RZ72	Actin-related protein 2/3 complex subunit 4	B2rz72	2.0E7	1.7E7	1.0E7	**1.6E7**	7.6E6	8.3E6	7.5E6	1.0E7	5.0E6	**7.7E6**	0.016	**-2.0**
Q66X93	Staphylococcal nuclease domain-containing protein 1	Snd1	2.3E7	1.9E7	1.4E7	**1.9E7**	9.3E6	1.1E7	7.4E6	1.4E7	3.4E6	**9.2E6**	0.025	**-2.0**
A0A0G2JZH3	Thrombospondin 3	A0a0g2jzh3	5.8E7	5.2E7	6.4E7	**5.8E7**	2.3E7	3.4E7	2.0E7	4.7E7	1.7E7	**2.8E7**	0.009	**-2.1**
A0A0G2JWX4	Keratin, type II cytoskeletal 2 epidermal	A0a0g2jwx4	3.5E7	2.1E7	1.9E7	**2.5E7**	1.8E7	1.7E7	1.3E7	3.9E6	9.5E6	**1.2E7**	0.045	**-2.1**
Q6PDV8	RCG31311	Q6pdv8	3.0E7	4.1E7	1.6E7	**2.9E7**	1.6E7	1.0E7	1.7E7	1.7E7	8.6E6	**1.4E7**	0.042	**-2.1**
I6L9G5	Rcn3 protein	I6l9g5	1.9E7	2.3E7	1.2E7	**1.8E7**	9.2E6	6.9E6	7.5E6	1.6E7	3.8E6	**8.6E6**	0.032	**-2.1**
Q05175	Brain acid soluble protein 1	Basp1	5.0E6	7.5E6	3.9E6	**5.5E6**	2.3E6	2.1E6	4.3E6	2.9E6	1.2E6	**2.6E6**	0.031	**-2.1**
F1LW91	Nuclear mitotic apparatus protein 1	F1lw91	3.1E6	2.4E6	2.5E6	**2.7E6**	9.8E5	1.1E6	2.5E6	1.4E6	1.9E5	**1.2E6**	0.035	**-2.1**
M0RA79	Uncharacterized LOC691828	M0ra79	1.0E8	9.3E7	1.0E8	**1.0E8**	4.7E7	3.4E7	5.7E7	6.7E7	2.8E7	**4.7E7**	0.002	**-2.1**
P08289	Alkaline phosphatase, tissue-nonspecific isozyme	Ppbt	4.7E7	5.1E7	2.8E7	**4.2E7**	2.2E7	1.6E7	2.9E7	1.9E7	1.1E7	**1.9E7**	0.012	**-2.2**
D3Z9E1	Elastin microfibril interfacer 1	D3z9e1	1.7E7	1.7E7	8.0E6	**1.4E7**	3.9E6	7.3E6	8.4E6	7.4E6	4.8E6	**6.3E6**	0.022	**-2.2**
P85845	Fascin	Fscn1	1.2E7	1.1E7	9.5E6	**1.1E7**	6.7E6	7.0E6	5.2E6	5.1E6	0.0E0	**4.8E6**	0.015	**-2.2**
A0A0G2JST3	Keratin, type II cytoskeletal 1	A0a0g2jst3	1.2E8	1.4E8	6.8E7	**1.1E8**	1.3E7	5.0E7	8.8E7	4.2E7	5.2E7	**4.9E7**	0.036	**-2.2**
G3V7U4	Lamin-B1	G3v7u4	7.8E7	6.9E7	4.3E7	**6.4E7**	2.1E7	2.6E7	2.9E7	4.9E7	1.7E7	**2.8E7**	0.016	**-2.2**

D3ZK97	Histone H3	D3zk97	2.1E8	2.3E8	4.2E8	**2.9E8**	1.6E8	2.0E8	1.2E8	1.2E8	2.2E7	**1.2E8**	0.046	**-2.3**
A0A0G2K9Q9	Keratin, type I cytoskeletal 17	A0a0g2k9q9	3.2E7	3.6E7	1.5E7	**2.8E7**	1.4E7	8.7E6	1.6E7	1.7E7	4.3E6	**1.2E7**	0.036	**-2.3**
A0A0G2KAM4	Fascin	A0a0g2kam4	1.2E7	1.1E7	9.5E6	**1.1E7**	6.6E6	7.0E6	4.3E6	5.1E6	0.0E0	**4.6E6**	0.012	**-2.3**
Q6LED0	Histone H3.1	H31	2.1E8	2.0E8	4.2E8	**2.8E8**	1.5E8	1.9E8	1.2E8	1.2E8	2.2E7	**1.2E8**	0.049	**-2.3**
D3ZJ08	Histone H3	D3zj08	2.4E8	2.2E8	4.2E8	**3.0E8**	1.6E8	1.9E8	1.2E8	1.2E8	2.0E7	**1.2E8**	0.029	**-2.4**
F1M013	60S ribosomal protein L7a	F1m013	3.3E7	3.2E7	2.2E7	**2.9E7**	1.4E7	1.1E7	3.9E6	1.8E7	1.2E7	**1.2E7**	0.005	**-2.5**
D3ZKU5	Similar to ribosomal protein L31	D3zku5	2.7E7	1.8E7	1.5E7	**2.0E7**	6.4E6	1.1E7	1.0E7	5.1E6	6.4E6	**7.8E6**	0.007	**-2.6**
F1LV13	Heterogeneous nuclear ribonucleoprotein M	F1lv13	1.4E7	1.6E7	8.2E6	**1.3E7**	4.1E6	4.3E6	3.8E6	9.1E6	3.1E6	**4.9E6**	0.012	**-2.6**
Q6IFV4	Keratin, type I cytoskeletal 13	K1c13	3.6E7	3.9E7	2.1E7	**3.2E7**	1.3E7	8.5E6	1.8E7	1.7E7	4.9E6	**1.2E7**	0.010	**-2.6**
F1LQ14	60S ribosomal protein L34	F1lq14	1.6E7	1.9E7	1.3E7	**1.6E7**	5.5E6	5.5E6	6.0E6	1.3E7	0.0E0	**6.0E6**	0.017	**-2.6**
D3ZBN0	Histone H1.5	H15	4.2E7	4.5E7	3.2E7	**4.0E7**	9.3E6	1.4E7	1.3E7	2.7E7	7.3E6	**1.4E7**	0.004	**-2.8**
G3V9R8	Heterogeneous nuclear ribonucleoprotein C	Hnrpc	1.2E7	4.9E6	6.2E6	**7.7E6**	4.6E6	3.0E6	0.0E0	4.1E6	2.1E6	**2.7E6**	0.042	**-2.8**
P02793	Ferritin light chain 1	Fril1	7.7E7	7.6E7	3.1E7	**6.1E7**	3.6E7	2.7E7	1.1E7	2.3E7	8.9E6	**2.1E7**	0.020	**-2.9**
A0A0G2JZS9	Uncharacterized protein	A0a0g2jzs9	7.8E7	4.2E7	7.4E7	**6.5E7**	0.0E0	2.4E7	3.1E7	3.4E7	1.5E7	**2.1E7**	0.009	**-3.1**
O35244	Peroxiredoxin-6	Prdx6	1.1E7	1.2E7	7.0E6	**9.9E6**	6.3E6	0.0E0	4.8E6	4.2E6	0.0E0	**3.1E6**	0.015	**-3.2**
A0A096MJE0	Keratin 32 (Fragment)	A0a096mje0	8.1E7	7.5E7	3.2E7	**6.3E7**	2.0E7	1.7E7	4.2E7	1.7E7	0.0E0	**1.9E7**	0.022	**-3.3**
A0A0H2UHU0	40S ribosomal protein S25	A0a0h2uhu0	2.9E7	2.3E7	1.1E7	**2.1E7**	1.0E7	0.0E0	4.7E6	1.4E7	3.3E6	**6.4E6**	0.026	**-3.3**
Q6IFU9	Keratin 16	Q6ifu9	7.7E7	6.9E7	7.9E7	**7.5E7**	1.9E7	1.1E7	3.1E7	3.1E7	1.6E7	**2.2E7**	0.0001	**-3.5**
Q5RKG9	Eukaryotic translation initiation factor 4B	Q5rkg9	7.8E5	1.4E6	8.1E5	**1.0E6**	3.5E5	5.2E5	0.0E0	5.4E5	0.0E0	**2.8E5**	0.018	**-3.6**
A0A0G2K531	Glutathione peroxidase	A0a0g2k531	2.7E7	1.2E7	1.4E7	**1.8E7**	1.1E7	8.3E6	0.0E0	0.0E0	4.8E6	**4.7E6**	0.027	**-3.7**
D3ZZN4	Uncharacterized protein	D3zzn4	1.3E7	1.4E7	9.4E6	**1.2E7**	5.7E6	0.0E0	8.4E6	0.0E0	1.8E6	**3.2E6**	0.010	**-3.9**
A0A0G2JSL0	Proteasome subunit beta type	A0a0g2jsl0	1.1E7	1.4E7	6.5E6	**1.0E7**	5.0E6	4.6E6	0.0E0	0.0E0	3.0E6	**2.5E6**	0.009	**-4.1**
P52296	Importin subunit beta-1	Imb1	1.2E7	1.0E7	6.3E6	**9.6E6**	5.3E6	0.0E0	0.0E0	2.6E6	3.2E6	**2.2E6**	0.007	**-4.3**
B5DF91	ELAV-like protein 1	Elav1	1.0E7	9.9E6	3.9E6	**7.9E6**	0.0E0	0.0E0	3.1E6	5.4E6	0.0E0	**1.7E6**	0.025	**-4.6**
D4ABT8	Heterogeneous nuclear ribonucleoprotein U-like 2	D4abt8	2.5E6	3.3E6	1.7E6	**2.5E6**	0.0E0	0.0E0	1.1E6	1.5E6	0.0E0	**5.3E5**	0.013	**-4.7**

D3Z9G3	Uncharacterized protein	D3z9g3	5.1E7	6.4E7	2.6E7	**4.7E7**	1.9E7	2.2E7	0.0E0	8.6E6	0.0E0	**9.9E6**	0.012	**-4.7**
G3V9T1	Cysteine-rich secretory protein LCCL domain-containing 2	G3v9t1	4.8E6	4.8E6	2.4E6	**4.0E6**	2.3E6	0.0E0	1.9E6	0.0E0	0.0E0	**8.5E5**	0.013	**-4.7**
P62083	40S ribosomal protein S7	Rs7	2.4E7	3.6E7	7.1E6	**2.2E7**	9.9E6	8.0E6	0.0E0	4.9E6	0.0E0	**4.6E6**	0.037	**-4.9**
D3ZN79	Similar to 60S ribosomal protein L35	D3zn79	3.7E7	5.0E7	2.3E7	**3.7E7**	1.5E7	1.8E7	0.0E0	0.0E0	2.9E6	**7.2E6**	0.009	**-5.1**
A0A0G2K9X1	Secreted phosphoprotein 24	A0a0g2k9x1	1.4E7	1.8E7	4.4E6	**1.2E7**	0.0E0	8.8E6	0.0E0	2.5E6	0.0E0	**2.3E6**	0.038	**-5.3**
B2RYQ2	Serine/threonine-protein phosphatase 2A activator	B2ryq2	2.9E6	4.5E6	3.0E6	**3.5E6**	0.0E0	0.0E0	0.0E0	3.0E6	0.0E0	**6.0E5**	0.017	**-5.8**
D4A0L4	Y-box-binding protein 3	D4a0l4	5.6E6	4.6E6	8.8E6	**6.4E6**	0.0E0	1.9E6	0.0E0	3.5E6	0.0E0	**1.1E6**	0.007	**-5.8**
G3V6S1	PRKC apoptosis WT1 regulator protein	G3v6s1	2.9E6	3.5E6	1.6E6	**2.7E6**	9.2E5	0.0E0	1.3E6	0.0E0	0.0E0	**4.5E5**	0.007	**-6.0**
G3V6P6	RNA binding motif protein 3, isoform CRA_a	G3v6p6	3.4E7	2.8E7	1.8E7	**2.7E7**	1.2E7	0.0E0	0.0E0	8.7E6	0.0E0	**4.2E6**	0.004	**-6.4**
D3ZFY8	Ubiquitin-conjugating enzyme E2 variant 1-like	D3zfy8	8.3E6	9.6E6	2.3E6	**6.7E6**	5.8E5	0.0E0	0.0E0	3.7E6	0.0E0	**8.6E5**	0.022	**-7.8**
F1LPR6	Immunoglobulin heavy constant mu	F1lpr6	1.5E7	1.6E7	2.5E7	**1.9E7**	3.0E6	0.0E0	0.0E0	4.9E6	2.7E6	**2.1E6**	0.001	**-8.9**
Q6AZ26	C-terminal binding protein 1	Q6az26	3.6E6	2.4E6	2.0E6	**2.7E6**	1.4E6	0.0E0	0.0E0	0.0E0	0.0E0	**2.7E5**	0.003	**-9.9**
P62275	40S ribosomal protein S29	Rs29	8.6E6	7.7E6	3.5E6	**6.6E6**	3.2E6	0.0E0	0.0E0	0.0E0	0.0E0	**6.3E5**	0.006	**-10.4**
B5DF03	Peptidylprolyl isomerase (Fragment)	B5df03	3.2E6	4.1E6	1.9E6	**3.1E6**	0.0E0	0.0E0	1.5E6	0.0E0	0.0E0	**2.9E5**	0.004	**-10.5**
B0BNA7	Eukaryotic translation initiation factor 3 subunit I	Eif3i	3.0E6	3.9E6	2.0E6	**3.0E6**	0.0E0	1.4E6	0.0E0	0.0E0	0.0E0	**2.8E5**	0.003	**-10.7**
Q5XIC1	Mannose-1-phosphate guanyltransferase alpha	Gmppa	2.3E6	3.5E6	0.0E0	**1.9E6**	0.0E0	0.0E0	0.0E0	0.0E0	0.0E0	**0.0E0**	0.043	**-1.9E6**
B2RZA9	Ube2l3 protein	B2rza9	5.0E6	4.3E6	0.0E0	**3.1E6**	0.0E0	0.0E0	0.0E0	0.0E0	0.0E0	**0.0E0**	0.035	**-3.1E6**
A0A140TAA4	Programmed cell death 6-interacting protein	A0a140taa4	3.9E6	4.3E6	2.1E6	**3.4E6**	0.0E0	0.0E0	0.0E0	0.0E0	0.0E0	**0.0E0**	0.001	**-3.4E6**
M0RD20	Calpain small subunit 1	M0rd20	5.6E6	5.0E6	0.0E0	**3.5E6**	0.0E0	0.0E0	0.0E0	0.0E0	0.0E0	**0.0E0**	0.034	**-3.5E6**
Q63002	Mannose 6-phosphate/insulin-like growth factor II receptor	Q63002	5.4E6	5.2E6	0.0E0	**3.5E6**	0.0E0	0.0E0	0.0E0	0.0E0	0.0E0	**0.0E0**	0.034	**-3.5E6**
Q4KM65	Cleavage and polyadenylation specificity factor subunit 5	Cpsf5	6.7E6	5.7E6	0.0E0	**4.1E6**	0.0E0	0.0E0	0.0E0	0.0E0	0.0E0	**0.0E0**	0.035	**-4.1E6**
